# Replicated associations of *TNFAIP3*, *TNIP1 *and *ETS1 *with systemic lupus erythematosus in a southwestern Chinese population

**DOI:** 10.1186/ar3514

**Published:** 2011-11-16

**Authors:** Hua Zhong, Xiao-lan Li, Ming Li, Li-xia Hao, Rong-wei Chen, Kun Xiang, Xue-bin Qi, Runlin Z Ma, Bing Su

**Affiliations:** 1 State Key Laboratory of Genetic Resources and Evolution, Kunming Institute of Zoology, Chinese Academy of Sciences, Kunming, Yunnan 650223, China; 2 Department of Dermatology and Rheumatology, The Affiliated Yan'an Hospital of Kunming Medical University, Kunming, Yunnan 650051, China; 3 Kunming Medical University, Kunming, Yunnan 650500, China; 4 State Key Laboratory of Molecular Developmental Biology, Institute of Genetics and Developmental Biology, Chinese Academy of Sciences, Beijing 100101, China; 5 Graduate School of Chinese Academy of Sciences, Beijing 100080, China

## Abstract

**Introduction:**

Recent genome-wide and candidate gene association studies in large numbers of systemic lupus erythematosus (SLE) patients have suggested approximately 30 susceptibility genes. These genes are involved in three types of biological processes, including immune complex processing, toll-like receptor function and type I interferon production, and immune signal transduction in lymphocytes, and they may contribute to the pathogenesis of SLE. To better understand the genetic risk factors of SLE, we investigated the associations of seven SLE susceptibility genes in a Chinese population, including *FCGR3A*, *FCGR2A*, *TNFAIP3*, *TLR9*, *TREX1*, *ETS1 *and *TNIP1*.

**Methods:**

A total of 20 SNPs spanning the seven SLE susceptibility genes were genotyped in a sample of 564 unrelated SLE patients and 504 unrelated healthy controls recruited from Yunnan, southwestern China. The associations of SNPs with SLE were assessed by statistical analysis.

**Results:**

Five SNPs in two genes (*TNFAIP3 *and *ETS1*) were significantly associated with SLE (corrected *P *values ranging from 0.03 to 5.5 × 10^-7^). Through stratified analysis, *TNFAIP3 *and *ETS1 *showed significant associations with multiple SLE subphenotypes (such as malar rash, arthritis, hematologic disorder and antinuclear antibody) while *TNIP1 *just showed relatively weak association with onset age. The associations of the SNPs in the other four genes were not replicated.

**Conclusions:**

The replication analysis indicates that *TNFAIP3*, *ETS1 *and *TNIP1 *are probably common susceptibility genes for SLE in Chinese populations, and they may contribute to the pathogenesis of multiple SLE subphenotypes.

## Introduction

Systemic lupus erythematosus (SLE) is a chronic autoimmune disease with marked clinical heterogeneity. The integral components of SLE pathology - including abnormal function of B cells and T cells that generates autoreactive lymphocytes and responses to many self-antigens, the perpetual production of a broad variety of autoantibodies that cause incomplete clearance and subsequent tissue deposition of immune complexes, the activation of complement and defective cellular apoptosis that generate a pool of potential autoantigens, and the expression of proinflammatory cytokines - all result in intense inflammation and multiple organ damage

Patients can be diagnosed as having SLE when they meet any four of 11 American College of Rheumatology classification criteria [1,2]. Genetic factors have been demonstrated to contribute to the susceptibility of SLE because of familial clustering [3-5] and increased concordance in monozygotic twins [6,7]. Since the 1970s, genetic analyses have been conducted to seek the susceptibility genes in SLE patients [8]. Especially, in the past 5 years, genome-wide association (GWA) and case-control studies in a large number of SLE patients have significantly expanded our understanding of the genetic basis of SLE [8-20]. Approximately 30 genes have been identified as playing important roles in SLE pathogenesis, and most of these genes have been shown to act in three types of biological processes: immune complex processing, toll-like receptor (TLR) function and type I interferon production, and immune signal transduction in lymphocytes [8,18,21,22]. These findings have revealed many robust associations and the related biological pathways involved in this complex disease. As an unusually heterogeneous disease, however, the genetic risk factors of SLE are yet to be fully dissected by conducting more studies, especially replications in populations with different ethnic backgrounds.

Among the top candidates for SLE susceptibility genes, Fcγ receptors (FCGRs) are involved in immune complex processing and are associated with SLE pathogenesis. Particularly, *FCGR2A *and *FCGR3A *possess functional variants, including missense mutations (*FCGR2A *H131R and *FCGR3A *V176F) that decrease their binding affinity to human IgG and affect the clearance of immune complexes [8,18,21,23-25]. Two missense mutations have been suggested to confer the increased risk for SLE [18,24,25] in spite of inconsistent results in different populations [26,27]

Type I interferon has been implicated in SLE pathophysiology, and there have been several genes identified in the related pathway [21]. The overproduction of type I interferon can promote the maturation of dendritic cells, and the expression of proinflammatory cytokines and chemokines, leading to diverse effects on immune functions including the activation of autoreactive B cells and T cells, the production of autoantibodies, and loss of self-tolerance [28,29].

TNFα-induced protein 3 (*TNFAIP3*), also known as the zinc-finger A20 protein, is a ubiquitin-editing enzyme that restricts both TNF receptor and TLR-induced NF-κB signals, and therefore is required for effective termination of NF-κB-mediated proinflammatory responses [30-32]. In recent GWA studies, genetic variants in the *TNFAIP3 *gene have been identified as contributing to the genetic risk of SLE in both European and Asian populations [12,15,19]. These variants include a potential causal SNP (rs2230926), which was suggested to reduce the ability of A20 to attenuate NF-κB signaling, resulting in a high level of type I interferon production [15,33].

An important category of innate immune receptors, TLRs can discriminate self-derived DNA from microbe-derived DNA. However, they can also be activated by host-derived nucleic acid; for example, nucleic acid-containing autoantibody complexes or apoptotic or necrotic cell debris, the deposition of which is a predominant feature of SLE disease [34,35]. Of the TLRs, TLR9 was suggested to be involved in the pathogenesis of SLE because its aberrant activation may lead to the production of type I interferon [28].

Another gene related to the DNA-recognition-activated type I interferon response pathway is the 3' repair exonuclease 1 (*TREX1*). Normally, *TREX1 *prevents activation of cell-intrinsic type I interferon response by digesting cytosolic single-stranded DNA [36]. When *TREX1 *is mutated, however, it will cause the activation of type I interferon and subsequent autoimmunity. Previous studies have shown that mutations in *TREX1 *were associated with SLE [37]; however, the association was inconsistent in different populations [38].

We conducted a case-control association study in 564 cases and 504 controls from Yunnan province of southwestern China. We screened a total of seven candidate genes, including *FCGR3A*, *FCGR2A*, *TNFAIP3*, *TLR9*, *TREX1 *and the two newly identified susceptibility genes E26 transformation-specific 1 (*ETS1*) and *TNFAIP3 *interacting protein 1 (*TNIP1*). The association of *ETS1 *and *TNIP1 *with SLE was first reported in a recent Chinese GWA study [19]. *TNIP1 *interacts with *TNFAIP3 *and may play a role in NF-κB inhibition together with *TNFAIP3*. The association of *TNIP1 *with SLE was also reported in the GWA study of Europeans [17]. *ETS1 *is a member of the E26 transformation-specific family of transcription factors that control a wide variety of cellular processes including immune cell differentiation and development, and was shown to negatively regulate T-helper type 17 (Th17) cell differentiation and terminal differentiation of B cells [39,40]. A recent study in Asian populations demonstrated that reduced expression of *ETS1 *may play a role in SLE pathogenesis through increased differentiation and activity of both plasma cells and Th17 cells [20]. Although the two new susceptibility genes were identified through GWA studies in large number of cases, disease susceptibility has to be validated via replication study in more populations. The aim of this replication study is to test the susceptibility of the seven genes for SLE in a southwestern Chinese population.

## Materials and methods

### Patients and controls

A total of 564 unrelated SLE patients (89.8% female and mean age 37.1 ± 13.6) and 504 unrelated healthy controls (87.5% female and mean age 39.3 ± 13.2) were recruited from 1999 to 2010 in Kunming, Yunnan, China. All SLE patients fulfilled the American College of Rheumatology classification criteria [1,2]. We obtained the detailed clinical data from 450 out of the 564 SLE patients, which were used for the stratified analysis. The protocol of the present study was approved by the Institutional Review Board of Kunming Institute of Zoology, Chinese Academy of Sciences. Informed consent was obtained from all participants.

### SNP selection

SNP selection was based on the published data [15,17-20,23-25,37,41] and the linkage disequilibrium patterns in the Han Chinese from Beijing plus Japanese population in the HapMap database (Figure S1 in Additional file 1). The SNPs rs11581823 and rs7539036 were selected for *FCGR3A*; rs10800309, rs4656308 and rs1801274 for *FCGR2A*; rs5029924, rs5029936, rs5029937, rs2230926 and rs610604 for *TNFAIP3*; rs187084, rs352140 and rs352162 for *TLR9*; rs2242150 and rs3135941 for *TREX1*; rs6590330 and rs4937333 for *ETS1*; and rs13168551, rs7708392 and rs10036748 for *TNIP1*. A total of 20 SNPs were selected (Table 1).

**Table 1:** SNP association analysis of the 18 SNPs in all cases and controls

Gene and SNP	**Position**^**a**^	Minor allele	Minor allele frequency		*P *value	**Corrected *P *value**^**b**^	Odds ratio (95% confidence interval)
						
			Case	Control			
*FCGR3A*							
rs11581823	1:161517384	C	0.0830	0.0975	0.28		0.84 (0.62 to 1.14)
rs7539036	1:161512731	T	0.0580	0.0714	0.25		0.80 (0.56 to 1.14)
*FCGR2A*							
rs4656308	1:161478751	C	0.1602	0.1456	0.36		1.12 (0.88 to 1.42)
rs1801274	1:161479745	G	0.3565	0.34	0.43		1.08 (0.90 to 1.29)
*TNFAIP3*							
rs5029924	6:138187498	T	0.0817	0.0470	**0.0017**	**0.030**	1.80 (1.25 to 2.61)
rs5029937	6:138195151	T	0.0812	0.0448	**0.0006**	**0.012**	1.88 (1.30 to 2.73)
rs2230926	6:138196066	G	0.0811	0.0448	**0.0007**	**0.012**	1.88 (1.30 to 2.72)
rs610604	6:138199417	G	0.0989	0.0984	1.00		1.00 (0.75 to 1.34)
*TLR9*							
rs187084	3:52261031	C	0.3773	0.3722	0.82		1.02 (0.85 to 1.22)
rs352140	3:52256697	A	0.3686	0.3635	0.82		1.02 (0.86 to 1.22)
rs352162	3:52252969	G	0.3850	0.3770	0.72		1.04 (0.87 to 1.24)
*TREX1*							
rs2242150	3:48505964	A	0.3535	0.3340	0.35		1.09(0.91 to 1.31)
rs3135941	3:48507667	C	0.0326	0.0392	0.47		0.82 (0.52 to 1.32)
*ETS1*							
rs6590330	11:128311059	A	0.4258	0.3086	**3.0 **× **10^-8^**	**5.5 × 10^-7^**	1.66 (1.39 to 1.99)
rs4937333	11:128330520	T	0.4814	0.3652	**9.3 × 10^-8^**	**1.7 × 10^-6^**	1.61 (1.35 to 1.92)
*TNIP1*							
rs13168551	5:150462638	T	0.2211	0.2505	0.14		0.85 (0.69 to 1.05)
rs7708392	5:150457485	G	0.2132	0.2587	**0.02**	0.39	0.78 (0.63 to 0.96)
rs10036748	5:150458146	C	0.2196	0.2552	0.08		0.82 (0.66 to 1.02)

Bold data are significant. **^a^**Genomic positions from NCBI Genome Build 37.1 (GRCh37) [56]. ^b^*P *values calculated by Fisher's exact test and then corrected by the Bonferroni criterion.

### Genotyping

Venous blood was collected from all participants, and genomic DNA was extracted using the standard phenol-chloroform method. DNA samples of the cases and controls were randomly distributed in the DNA plates. All of the selected SNPs were genotyped by the SNaPShot method on an ABI 3130 genetic analyzer (Applied Biosystems, Foster City, CA, USA). The SNP genotype callings were automatically performed using ABI GeneMapper 4.0 (Applied Biosystems, Foster City, CA, USA) and verified manually. To ensure the accuracy of genotyping, we conducted bidirectional sequencing on 32 randomly selected individuals; no genotyping errors were found. The genotyping success rate for the selected SNPs is 96.7%.

### Statistical analysis

Hardy-Weinberg equilibrium and allelic and genotypic associations were performed in 564 cases and 504 controls using PLINK 1.07 [42]. We also conducted the association analysis in females only by considering sex as a covariate. The resulting *P *values were corrected by applying the Bonferroni criterion, in which *P *values were multiplied by the number of tested SNPs to produce the Bonferroni-corrected *P *values. The power to detect association was calculated using the CaTs program [43,44], based on the minor allele frequency of each SNP in our case-control samples and the effect size calculated in the allelic association analysis. The prevalence of 0.06 was used according to the literature data [45,46].

To further investigate whether certain SNPs exclusively confer the genetic risk in one or more specific subphenotypes and to discover potential association of certain SNPs with SLE disease, stratified analysis based on onset age (age 27 was used, according to the literature data [45-47]) and 11 American College of Rheumatology-defined clinical subphenotypes were conducted, in which association was assessed by comparing allele and genotype distributions between patients with a certain subphenotype and controls, between patients without the subphenotype and controls, and between patients with and without the subphenotype (paired patient groups). Often, the associations can be observed in both of certain paired patient groups. We therefore compared the frequencies of minor alleles in controls and paired patient groups (assuming that minor alleles should be significantly enriched in a certain subphenotype if they really contribute to the subphenotype pathogenesis), and calculated the conditional frequencies of onset age and other subphenotypes in the paired patient groups to understand their compositions and to re-evaluate certain associations detected in the stratified analysis.

## Results

All SNPs were in Hardy-Weinberg equilibrium in both cases and controls (*P *> 0.05 after Bonferroni correction) except for rs10800309 in controls (*P *= 1.69 × 10^-9^) and rs5029936 in cases (*P *= 8.17 × 10^-4^) - these two SNPs were therefore excluded, resulting in a total of 18 SNPs for subsequent association analysis. According to the obtained clinical information, a total of 450 SLE patients have the complete data for the 11 American College of Rheumatology clinical subphenotypes, including malar rash (72.9%), discoid rash (16.2%), photosensitivity (17.3%), oral ulcers (17.1%), arthritis (75.8%), serositis (33.8%), renal disorder (60.7%), neurologic disorder (11.1%), hematologic disorder (82.2%), immunologic disorder (75.6%) and antinuclear antibody (97.1%).

### Association of *TNFAIP3*, *ETS1 *and *TNIP1 *with SLE

The result of allelic association for each SNP is shown in Table 1. A total of six SNPs were found to be significantly related to SLE (*P *< 0.05, Table 1), and five of them remained significant after Bonferroni correction, including three SNPs in *TNFAIP3 *(rs5029924, rs5029937 and rs2230926) and two SNPs in *ETS1 *(rs6590330 and rs4937333). The SNP rs7708392 in *TNIP1 *showed weak association (*P *= 0.0211, uncorrected), but became nonsignificant after correction. Association analysis using the genotype data generated similar results (Table S1 in Additional file 1). The results remained the same when only females were considered (data not shown).

### Lack of association of *FCGR3A*, *FCGR2A*, *TLR9 *and *TREX1 *with SLE

Significant association with SLE was not observed in the selected SNPs for *FCGR3A*, *FCGR2A*, *TLR9 *and *TREX1 *in either the allelic or the genotypic association analyses (Table 1; see also Table S1 in Additional file 1). The same results were obtained when only considering the females (data not shown).

The power calculation was performed (Table S2 in Additional file 1) to detect false-positive and false-negative association results. For SNPs rs5029924, rs5029937 and rs2230926 in *TNFAIP3*, rs6590330 and rs4937333 in *ETS1*, and rs7708392 in *TNIP1*, the sample size of this study showed relatively high power (76 to 100%) to detect association when allowing a false-positive rate of 0.05; however, the sample size showed rather low power for the other SNPs (0 to 57%). It is noteworthy that SNPs rs610604 (in *TNFAIP3*), rs187084, rs352140 and rs352162 (in *TLR9*) showed a rather low effect size (odds ratio (OR) = 1.00 to 1.04), implying that they have almost no or a rather weak contribution to SLE pathogenesis in the Chinese population. As for the SNPs in *FCGR2A *and *TREX1*, although no significant association was detected (probably due to the small sample size), the same trend was observed as reported in previous studies [14,18,37].

### Stratified analysis

**Figure 1:**
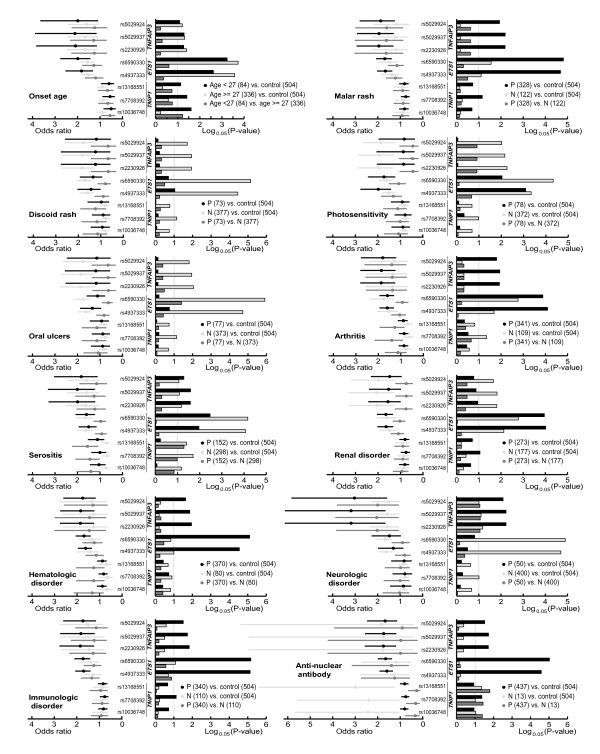
**Associations of *TNFAIP3*, *ETS1 *and *TNIP1 *with systemic lupus erythematosus assessed by stratified analysis**. P, patient group with a certain subphenotype; N, patient group without the subphenotype. Number in parentheses is the sample size. Vertical dashed line in each subphenotype panel indicates the cutoff value of statistical significance (*P *= 0.05).

As shown in Figure 1 (see also Table S3 in Additional file 1), the SNPs rs5029924, rs5029937 and rs2230926 in *TNFAIP3 *were observed to be significantly associated with onset age and most of the clinical subphenotypes, including malar rash, arthritis, serositis, neurologic disorder, hematologic disorder, immunologic disorder and antinuclear antibody (OR = 1.67 to 3.18, *P *= 0.021 to 0.001). For *ETS1*, SNPs rs6590330 and rs4937333 showed significant association with onset age and multiple clinical subphenotypes, including malar rash, photosensitivity, arthritis, serositis, renal disorder, hematologic disorder, immunologic disorder and antinuclear antibody (OR = 1.50 to 1.98, *P *= 0.003 to 1.9 × 10^-7^). For *TNIP1*, the SNP rs7708392 was also observed to be associated with onset age as well as several subphenotypes, including malar rash, renal disorder, immunologic disorder and antinuclear antibody (OR = 0.75 to 0.76, *P *= 0.017 to 0.043). In addition, the minor allele (rs3135941C) in *TEX1 *showed a weak association with the subphenotype of neurologic disorder (with a zero frequency in this subphenotype, *P *= 0.042). The genotypic analysis with the subphenotypes generated similar results (Table S4 in Additional file 1).

As expected, certain SNPs also showed significance in both of the paired patient groups (Figure 1; see also Tables S3 and S4 in Additional file 1). Significance was observed for rs5029924, rs5029937 and rs2230926 (*TNFAIP3*) in the paired patient groups of onset age and subphenotypes of serositis and neurologic disorder; for rs6590330 and rs4937333 (*ETS1*) in the paired patient groups of onset age and subphenotypes of malar rash, photosensitivity, arthritis, serositis and renal disorder; and for rs7708392 (*TNIP1*) in the paired patient groups of antinuclear antibody (Figure 1). Interestingly, SNPs rs6590330 and rs4937333 (*ETS1*) showed significance in the patient groups without the subphenotypes of oral ulcers and neurologic disorder when compared with the patient groups with these subphenotypes.

**Figure 2:**
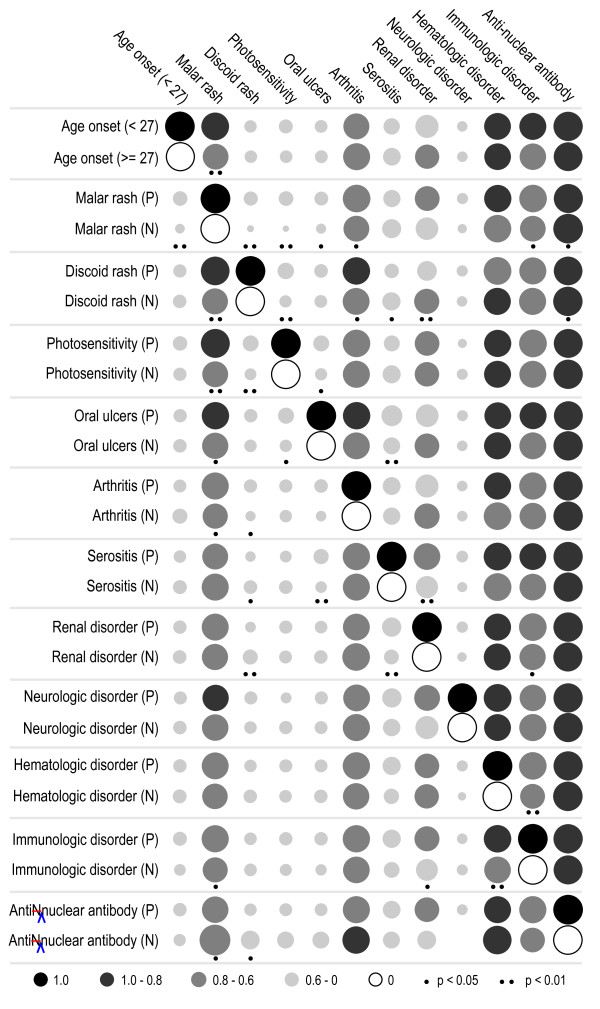
**Comparison of the composition of each patient group with or without a certain subphenotype**. P, patient group with a certain subphenotype; N, patient group without the subphenotype. The area of each cycle is proportional to the conditional frequency, which is the proportion of each subphenotype (upper side of figure) in a P or N group.

To discern between true and false associations, we compared the frequency distribution of the minor allele (only for *TNFAIP3*, *ETS1 *and *TNIP1*) in controls and the paired patient groups based on ORs (Figure 1) and compared the composition of these paired patient groups by calculating the conditional frequencies of onset age and other subphenotypes (Figure 2). Risk alleles of *TNFAIP3 *and *ETS1 *were found to be significantly enriched in subphenotypes of malar rash, arthritis, hematologic, neurologic and subphenotype antinuclear antibody - with the highest OR when comparing the patient group with the subphenotype with the controls, and the lowest OR when comparing the patient group without the subphenotype with the controls. Likewise, protective alleles of *TNIP1 *were found to have a similar enrichment pattern when considering onset age. We believe that these associations are probably true positive. As for the comparison of the composition of paired groups shown in Figure 2, most of them are similar. Although only a few subphenotypes showed the difference of conditional frequency, they would help excluding the false positive. For example, *TNFAIP3 *showed significance and the enrichment of minor allele in both of the paired groups for onset age, which have different frequencies in malar rash-positive patients (*P *< 0.01). Since the malar rash subphenotype was significantly related with *TNFAIP3*, its decreased frequency in patients aged ≥ 27 reduced the corresponding effect size, suggesting that the association of *TNFAIP3 *with onset age was probably caused by other subphenotypes associated with *TNFAIP3*. Another example is that *ETS1 *showed significance and a larger effect size in patient groups without discoid rash, which was probably due to the significantly increased frequency of antinuclear antibody (Figure 2).

## Discussion

Among the seven genes tested for SLE susceptibility, three (*TNFAIP3*, *TNIP1 *and *ETS1*) were successfully replicated for the associations. Consistent with recent studies [19,20,48-50], rs2230926 in *TNFAIP3*, rs7708392 in *TNIP1*, and rs4937333 and rs6590330 in *ETS1 *showed significant associations with SLE (Table 1; see also Table S1 in Additional file 1). The SNP rs2230926 in *TNFAIP3 *is a nonsynonymous variant, and the risk allele (rs2230926G) was suggested to downregulate the expression and activity of inhibiting the TNF-induced NF-κB activity of A20 protein [15]. A recent study demonstrated that the functional variant TT > A was significantly associated with SLE in European and Korean populations, leading to reduced *TNFAIP3 *mRNA and A20 protein expression [33]. Similarly, a functional SNP (rs1128334), tightly linked with rs6590330 and located in the 3' UTR of *ETS1*, was significantly associated with lower expression of *ETS1 *in SLE patients (*P *< 0.0001) [20]. Since *ETS1 *is a negative regulator for the differentiation of both Th17 cells and B cells, the decreased expression of *ETS1 *possibly contributes to the SLE pathogenesis through the increased differentiation and activity of both plasma cells and Th17 cells [20,39,40]. Combined with the reported data and the power calculation (Table S2 in Additional file 1), our results support *TNFAIP3 *and *ETS1 *as probable common genetic risk factors for SLE in different populations, highlighting the importance of two biological pathways: type I interferon (involving *TNFAIP3*) and immune signal transduction in lymphocytes (involving *ETS1*).

The observed association for *TNIP1 *in our samples was weak. This gene was recently identified in European (rs7708392) and Chinese (rs10036748) populations via GWA studies [17,19], and its association with SLE has been replicated in Japanese (rs7708392) and Han Chinese (rs10036748) populations [48,51]. In our samples, the G allele of rs7708392 showed a protective effect for SLE (allele frequency in SLE: 21.3%, control: 25.9%; OR = 0.78, 95% confidence interval = 0.63 to 0.96, *P *= 0.02), which is similar to the results reported in a Japanese population [48]. Interestingly, the G allele frequencies of rs7708392 are highly different between Asian (~30%) and European (~70%) populations, suggesting genetic heterogeneity at this locus among different populations. Although the selected SNPs in *TNIP1 *showed weak associations with SLE, they have a very similar minor allele frequency distribution to a recent Chinese GWA study (rs10036748: 20.5% in cases and 25.4% in controls, OR = 0.80) [19]. The power calculation (76%) suggested the reliability of the association of *TNIP1 *with SLE and also indicated that a larger sample size could help further confirm the role of *TNIP1 *in Chinese SLE patients.

We did not replicate the associations of the other four genes (*FCGR3A*, *FCGR2A*, *TLR9 *and *TREX1*) in our samples (Table 1; see also Table S1 in Additional file 1). *FCGR3A *and *FCGR2A *are involved in the clearance of immune complexes, and *TLR9 *and *TREX1 *may promote the production of type I interferon [28,36]. The previously proposed SLE susceptibility of these four genes was inconsistent among different populations [26,27,38,41]. No association was reported in Chinese, Korean and Japanese populations [26,38,41,52], although weak associations of several SNPs were observed; for example, rs396991 in *FCGR3A *[53], rs1801274 in *FCGR2A *[54], rs352140 in *TLR9 *[55] and the *TREX1 *polymorphism -20260G > C [38]. Hence, whether these four genes contribute to SLE susceptibility calls for more studies, as implied in the power calculation results (Table S2 in Additional file 1).

In the stratified analysis, *TNFAIP3*, *ETS1 *and *TNIP1 *remained the significant associations with SLE (Figure 1; see also Tables S3 and S4 in Additional file 1). *TNFAIP3 *and *ETS1 *were significantly associated with most subphenotypes, but none of them showed association with oral ulcers (Figure 1). As for *TNIP1*, the minor allele of rs7708392 was associated with a few SLE subphenotypes including malar rash, renal disorder and antinuclear antibody, which were also observed in the Japanese population [48]. It should be noted among several subphenotypes with high frequencies that malar rash, hematologic disorder, immunologic disorder and antinuclear antibody co-occurred in 48% of patients, and the last three subphenotypes showed a higher frequency of co-occurrence (62.67%). These features may cause false-positive associations as long as one of these co-occurred subphenotypes contributes greatly to the pathogenesis of SLE. We should therefore be cautious in interpreting the association results of SLE subphenotypes, and functional data are needed to confirm the contributions of the risk genes to the SLE pathogenesis.

## Conclusions

Our replication study confirmed the association of *TNFAIP3*, *ETS1 *and *TNIP1 *with SLE susceptibility in a southwestern Chinese population. In particular, *TNFAIP3 *and *ETS1 *are probably common genetic risk factors for SLE among Asian populations, and they contribute to most of the clinical subphenotypes of SLE, supporting the critical roles of two recently proposed biological pathways: type I interferon and immune signal transduction in lymphocytes in the pathogenesis of SLE.

## Abbreviations

ETS1: E26 transformation-specific 1; FCGR: Fcγ receptor; GWA: genome-wide association; NF: nuclear factor; OR: odds ratio; SLE: systemic lupus erythematosus; SNP: single nucleotide polymorphism; Th: T-helper type; TLR: toll-like receptor; TNF: tumor necrosis factor; TNFAIP3: TNFα-induced protein 3; TNIP1: TNFAIP3 interacting protein 1; TREX1: the 3' repair exonuclease 1; UTR: untranslated region.

## Competing interests

The authors declare that they have no competing interests.

## Authors' contributions

LX, ZH, MRZ and SB designed the study. ZH, LX, HL, CR, XK and QX carried out sample collection, DNA extraction and genotyping. LX, HL and CR collected clinical information. ZH, LX, LM, MRZ and SB performed data analysis and wrote the manuscript. All authors read and approved the final manuscript.

## Supplementary Material

Additional file 1 **SNP selection, analysis and distribution data**. Figure S1 shows gene structures and linkage disequilibrium plots calculated by Haploview 4.2 based on the HapMap Phase II dataset for the Han Chinese from Beijing plus Japanese (CHB + JPT) population. Table S1 presents genotype association analysis of the 18 SNPs in all cases and controls. Table S2 presents power analysis of the 18 SNPs in the present study. Table S3 presents the allele frequency distribution of the 18 SNPs in controls and different subphenotypes. Table S4 presents the genotype distribution of the 18 SNPs in controls and different subphenotypes. Click here for file
