# Supplementary material for: Replicated associations of TNFAIP3, TNIP1 and ETS1 with systemic lupus erythematosus in a southwestern Chinese population
Source: Arthritis Res Ther. 2011 Nov 16;13(6):R186. doi: 10.1186/ar3514 (PMC3334635; doi:10.1186/ar3514)
Supplement: Additional file 1 — SNP selection, analysis and distribution data. Figure S1 shows gene structures and linkage disequilibrium plots calculated by Haploview 4.2 based on the HapMap Phase II dataset for the Han Chinese from Beijing plus Japanese (CHB + JPT) population. Table S1 presents genotype association analysis of the 18 SNPs in all cases and controls. Table S2 presents power analysis of the 18 SNPs in the present study. Table S3 presents the allele frequency distribution of the 18 SNPs in controls and different subphenotypes. Table S4 presents the genotype distribution of the 18 SNPs in controls and different subphenotypes. [file ar3514-S1.PDF]

Figure S1. Gene structures and linkage disequilibrium plots calculated by Haploview 4.2 based on HapMap Phase II data set for CHB + JPT. LD blocks were defined by the algorithm "Confidence Intervals" (Gabriel et al, Science, 2002). D prime values were shown in LD blocks.

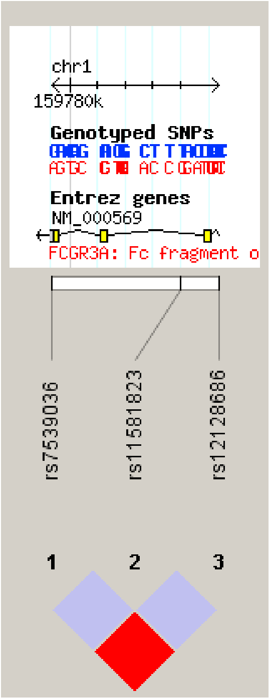

Figure S1a: Gene structure and linkage disequilibrium plot of *FCGR3A*. A functional mutation *FCGR3A* V176F (rs396991) was reported associated with SLE (ref.). According to the LD plot, we can see that three tagging SNPs were obtained from HapMap Phase II data set for CHB + JPT. Since rs7539036 and rs12128686 have tight linkage, finally, we selected rs7539036 and rs11581823 to detect the potential disease susceptibility of *FCGR3A* to SLE.

Ref: Edberg JC, Langefeld CD, Wu J, Moser KL, Kaufman KM, Kelly J, Bansal V, Brown WM, Salmon JE, Rich SS, Harley JB, Kimberly RP: **Genetic linkage and association of Fcgamma receptor IIIA (CD16A) on chromosome 1q23 with human systemic lupus erythematosus**. *Arthritis Rheum* 2002, **46**(8):2132-2140.

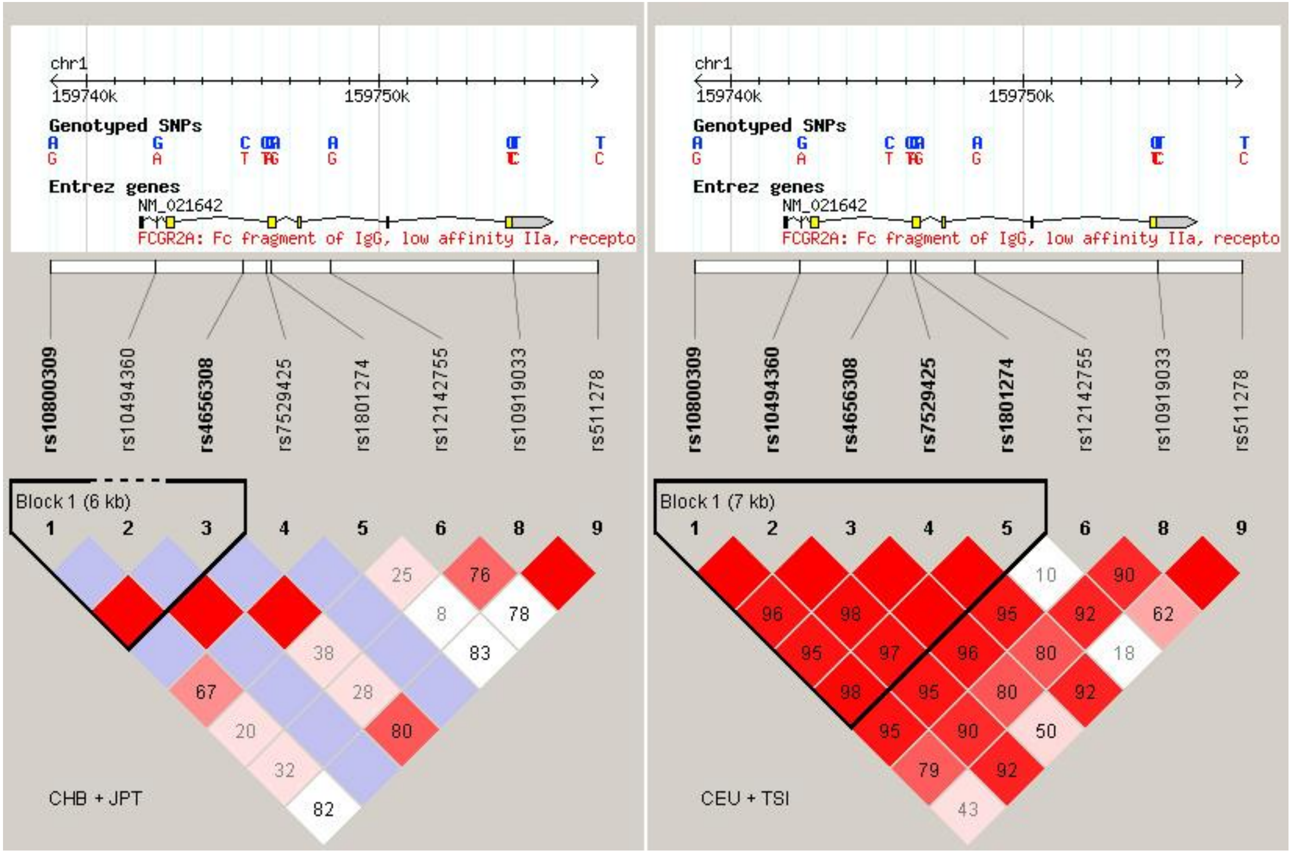

Figure S1b: Gene structure and linkage disequilibrium plot of *FCGR2A*. Rs10800309 and functional mutation *FCGR2A* H131R (rs1801274) were reported associated with SLE (ref.). So we replicated these two SNPs here. An additional marker rs4656308 between rs10800309 and rs1801274 was also included.

Ref: Harley JB, Alarcon-Riquelme ME, Criswell LA, Jacob CO, Kimberly RP, Moser KL, Tsao BP, Vyse TJ, Langefeld CD, Nath SK, Guthridge JM, Cobb BL, Mirel DB, Marion MC, Williams AH, Divers J, Wang W, Frank SG, Namjou B, Gabriel SB, Lee AT, Gregersen PK, Behrens TW, Taylor KE, Fernando M, Zidovetzki R, Gaffney PM, Edberg JC, Rioux JD, Ojwang JO *et al*: **Genome-wide association scan in women with systemic lupus erythematosus identifies susceptibility variants in ITGAM, PXK, KIAA1542 and other loci**. *Nat Genet* 2008, **40**(2):204-210.

Graham RR, Hom G, Ortmann W, Behrens TW: **Review of recent genome-wide association scans in lupus**. *J Intern Med* 2009, **265**(6):680-688.

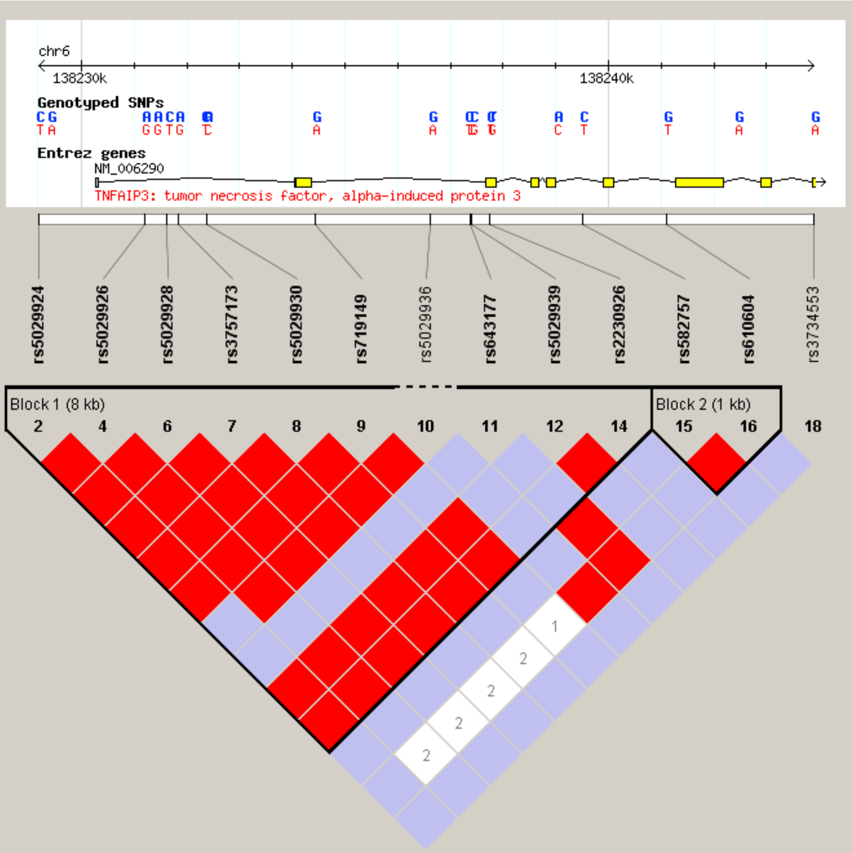

Figure S1c: Gene structure and linkage disequilibrium plot of *TNFAIP3*. Rs5029939 and rs2230926 were detected to be associated with SLE by GWAS (ref). As shown in this figure, these two tagging SNPs are closely linked. So we just selected rs2230926 (detected in two Chinese GWA studies) to perform this study. We also selected 4 additional SNPs (rs5029924, rs5029936, rs5029937 and rs610604), spanning block 1 and 2.

Ref: Graham RR, Cotsapas C, Davies L, Hackett R, Lessard CJ, Leon JM, Burtt NP, Guiducci C, Parkin M, Gates C, Plenge RM, Behrens TW, Wither JE, Rioux JD, Fortin PR, Graham DC, Wong AK, Vyse TJ, Daly MJ, Altshuler D, Moser KL, Gaffney PM: **Genetic variants near TNFAIP3 on 6q23 are associated with systemic lupus erythematosus**. *Nat Genet* 2008, **40**(9):1059-1061.

Musone SL, Taylor KE, Lu TT, Nititham J, Ferreira RC, Ortmann W, Shifrin N, Petri MA, Kamboh MI, Manzi S, Seldin MF, Gregersen PK, Behrens TW, Ma A, Kwok PY, Criswell LA: **Multiple polymorphisms in the TNFAIP3 region are independently associated with systemic lupus erythematosus**. *Nat Genet* 2008, **40**(9):1062-1064.

Han JW, Zheng HF, Cui Y, Sun LD, Ye DQ, Hu Z, Xu JH, Cai ZM, Huang W, Zhao GP, Xie HF, Fang H, Lu QJ, Li XP, Pan YF, Deng DQ, Zeng FQ, Ye ZZ, Zhang XY, Wang QW, Hao F, Ma L, Zuo XB, Zhou FS, Du WH, Cheng YL, Yang JQ, Shen SK, Li J, Sheng YJ *et al*: **Genome-wide association study in a Chinese Han population identifies nine new susceptibility loci for systemic lupus erythematosus**. *Nat Genet* 2009, **41**(11):1234-1237.

Yang W, Shen N, Ye DQ, Liu Q, Zhang Y, Qian XX, Hirankarn N, Ying D, Pan HF, Mok CC, Chan TM, Wong RW, Lee KW, Mok MY, Wong SN, Leung AM, Li XP, Avihingsanon Y, Wong CM, Lee TL, Ho MH, Lee PP, Chang YK, Li PH, Li RJ, Zhang L, Wong WH, Ng IO, Lau CS, Sham PC *et al*: **Genome-wide association study in Asian populations identifies variants in ETS1 and WDFY4 associated with systemic lupus erythematosus**. *PLoS Genet* 2010, **6**(2):e1000841.

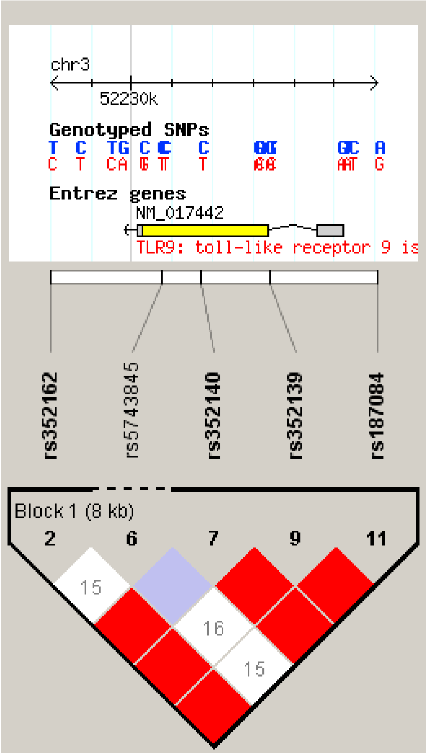

Figure S1d: Gene structure and linkage disequilibrium plot of *TLR9*. *TLR9* was suggested to be involved in the pathogenesis of SLE and certain SNPs were detected associated with SLE, for example, rs352140 in Chinese population (ref). As shown in this figure, this gene consists of one LD block in CHB + JPT population. So rs352140 can be used to investigate the association of *TLR9* and SLE. But, we still typed two additional SNPs, rs352162 and rs187084, spanning the whole gene.

Ref: Ardoin SP, Pisetsky DS: **Developments in the scientific understanding of lupus**. *Arthritis Res Ther* 2008, **10**(5):218.

Xu CJ, Zhang WH, Pan HF, Li XP, Xu JH, Ye DQ: **Association study of a single nucleotide polymorphism in the exon 2 region of toll-like receptor 9 (TLR9) gene with susceptibility to systemic lupus erythematosus among Chinese**. *Mol Biol Rep* 2009, **36**(8):2245-2248.

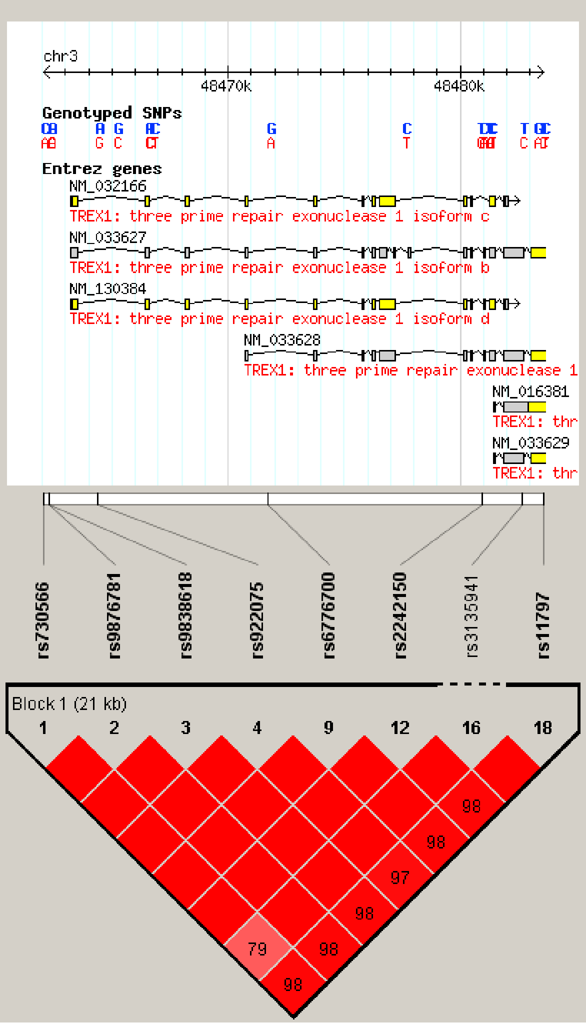

Figure S1e: Gene structure and linkage disequilibrium plot of *TREX1*. Normally, *TREX1* can prevent activation of cell-intrinsic type I interferon response by digesting cytosolic ssDNA and it was also reported that mutations in *TREX1* can cause the activation of type I interferon and subsequent autoimmunity (ref). As shown in this figure, tagging SNPs in *TREX1* form one LD block and SNPs at the left of rs3135941 have a complete linkage. So we selected rs2242150 to represent this region. Finally, rs2242150 and rs3135941 (5'- UTR) were selected.

Ref: Stetson DB, Ko JS, Heidmann T, Medzhitov R: **Trex1 prevents cell-intrinsic initiation of autoimmunity**. *Cell* 2008, **134**(4):587-598.

Lee-Kirsch MA, Gong M, Chowdhury D, Senenko L, Engel K, Lee YA, de Silva U, Bailey SL, Witte T, Vyse TJ, Kere J, Pfeiffer C, Harvey S, Wong A, Koskenmies S, Hummel O, Rohde K, Schmidt RE, Dominiczak AF, Gahr M, Hollis T, Perrino FW, Lieberman J, Hubner N: **Mutations in the gene encoding the 3'-5' DNA exonuclease TREX1 are associated with systemic lupus erythematosus**. *Nat Genet* 2007, **39**(9):1065-1067.

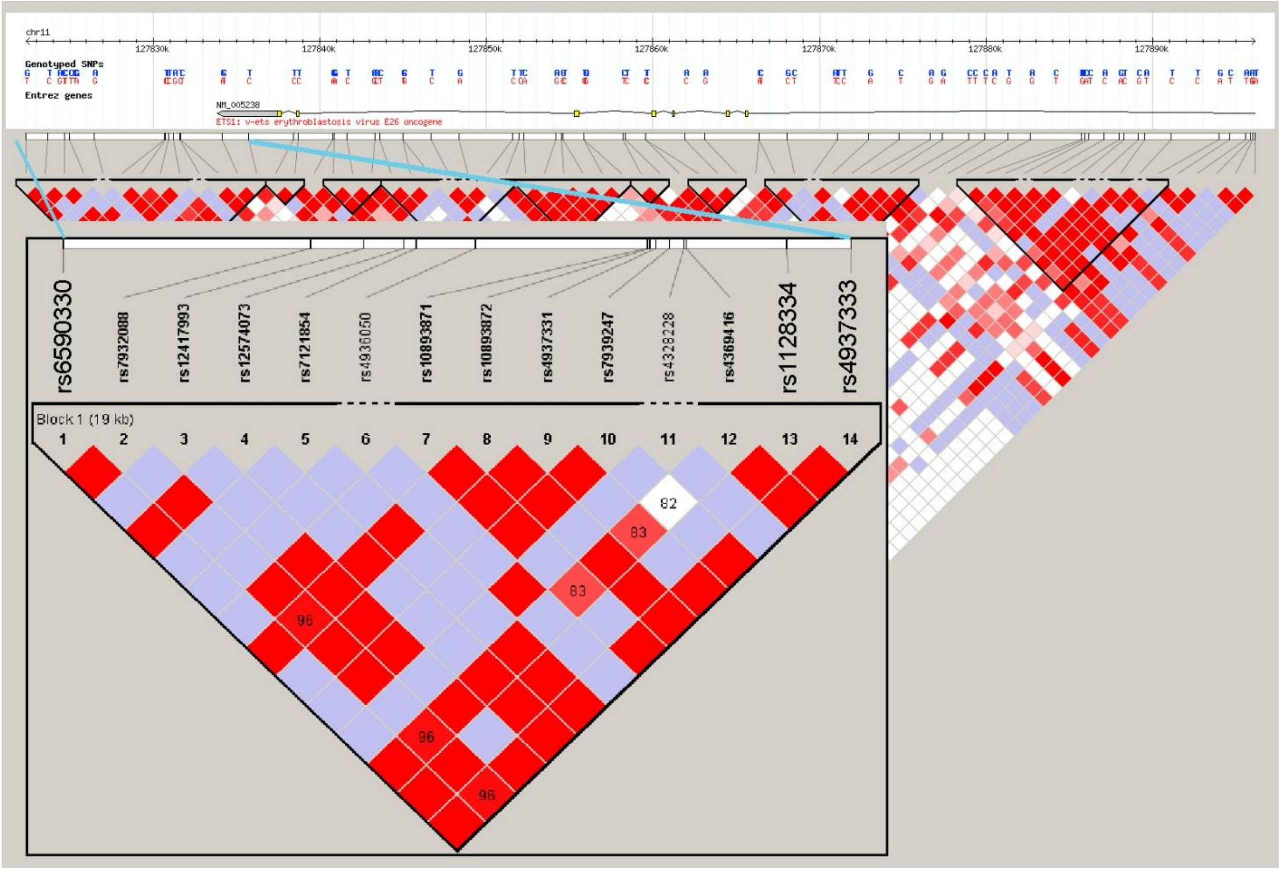

Figure S1f: Gene structure and linkage disequilibrium plot of *ETS1*. Three downstream SNPs (rs6590330, rs7932088 and rs10893872) and two 3'-UTR-located SNPs (rs1128334 and rs4937333) were reported significantly associated with SLE in Chinese GWA studies (ref). As shown in this figure, rs6590330 and rs7932088 are closely linked; rs10893872, rs1128334 and rs4937333 also have close linkage. So finally we selected rs6590330 and rs4937333 to perform this replication study.

Ref: Han JW, Zheng HF, Cui Y, Sun LD, Ye DQ, Hu Z, Xu JH, Cai ZM, Huang W, Zhao GP, Xie HF, Fang H, Lu QJ, Li XP, Pan YF, Deng DQ, Zeng FQ, Ye ZZ, Zhang XY, Wang QW, Hao F, Ma L, Zuo XB, Zhou FS, Du WH, Cheng YL, Yang JQ, Shen SK, Li J, Sheng YJ *et al*: **Genome-wide association study in a Chinese Han population identifies nine new susceptibility loci for systemic lupus erythematosus**. *Nat Genet* 2009, **41**(11):1234-1237.

Yang W, Shen N, Ye DQ, Liu Q, Zhang Y, Qian XX, Hirankarn N, Ying D, Pan HF, Mok CC, Chan TM, Wong RW, Lee KW, Mok MY, Wong SN, Leung AM, Li XP, Avihingsanon Y, Wong CM, Lee TL, Ho MH, Lee PP, Chang YK, Li PH, Li RJ, Zhang L, Wong WH, Ng IO, Lau CS, Sham PC *et al*: **Genome-wide association study in Asian populations identifies variants in ETS1 and WDFY4 associated with systemic lupus erythematosus**. *PLoS Genet* 2010, **6**(2):e1000841.

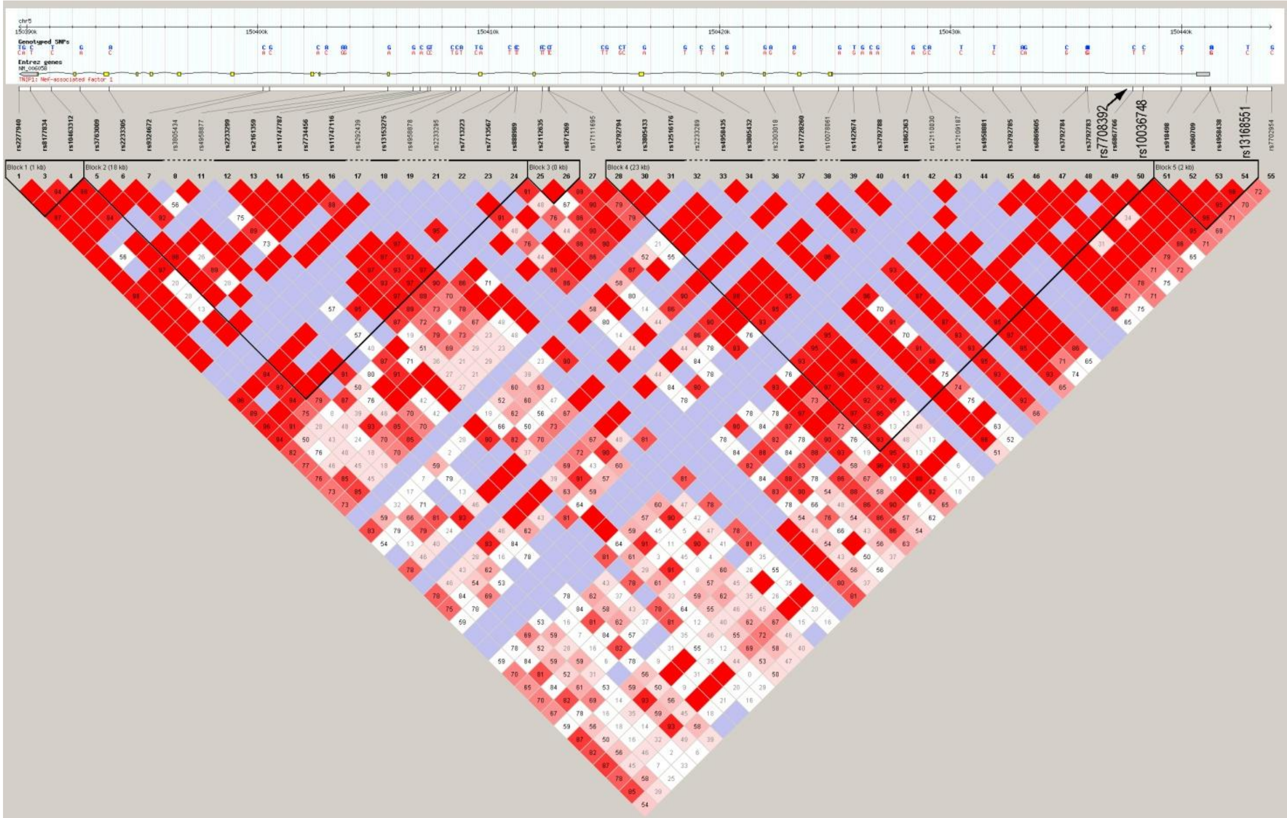

Figure S1g: Gene structure and linkage disequilibrium plot of *TNIP1*. Rs7708392 and rs10036748 were identified to be associated with SLE in Europeans and Chinese, respectively (ref). Rs7708392 (marked by arrow) is close to rs10036748, but it does not occur in this LD plot as a tagging SNP. We selected these two GWAS-derived tagging SNPs, together with an additional SNP rs13168551 (located in the 5'-upstream region of *TNIP1*) shown in LD block 5.

Ref: Gateva V, Sandling JK, Hom G, Taylor KE, Chung SA, Sun X, Ortmann W, Kosoy R, Ferreira RC, Nordmark G, Gunnarsson I, Svenungsson E, Padyukov L, Sturfelt G, Jonsen A, Bengtsson AA, Rantapaa-Dahlqvist S, Baechler EC, Brown EE, Alarcon GS, Edberg JC, Ramsey-Goldman R, McGwin G, Jr., Reveille JD, Vila LM, Kimberly RP, Manzi S, Petri MA, Lee A, Gregersen PK *et al*: **A large-scale replication study identifies TNIP1, PRDM1, JAZF1, UHRF1BP1 and IL10 as risk loci for systemic lupus erythematosus**. *Nat Genet* 2009, **41**(11):1228-1233.

Han JW, Zheng HF, Cui Y, Sun LD, Ye DQ, Hu Z, Xu JH, Cai ZM, Huang W, Zhao GP, Xie HF, Fang H, Lu QJ, Li XP, Pan YF, Deng DQ, Zeng FQ, Ye ZZ, Zhang XY, Wang QW, Hao F, Ma L, Zuo XB, Zhou FS, Du WH, Cheng YL, Yang JQ, Shen SK, Li J, Sheng YJ *et al*: **Genome-wide association study in a Chinese Han population identifies nine new susceptibility loci for systemic lupus erythematosus**. *Nat Genet* 2009, **41**(11):1234-1237.

Table S1. Genotype association analysis of the 18 SNPs in all cases and controls.

Gene	SNP	Genotype	Genotype counts		P	Corrected P †
			Case	Control		
FCGR3A	rs11581823	CC/CA/AA	0/89/447	1/91/385	0.28	
	rs7539036	TT/CT/CC	0/63/480	0/71/426	0.23	
FCGR2A	rs4656308	CC/CT/TT	19/136/388	12/121/365	0.55	
	rs1801274	GG/GA/AA	68/249/223	68/202/227	0.21	
TNFAIP3	rs5029924	TT/CT/CC	3/83/459	1/43/435	**0.003**	0.056
	rs5029937	TT/GT/GG	3/82/457	1/43/458	**0.001**	**0.026**
	rs2230926	GG/GT/TT	3/83/463	1/43/458	**0.001**	**0.025**
	rs610604	GG/GT/TT	10/88/448	4/90/404	0.27	
TLR9	rs187084	CC/CT/TT	87/235/220	65/231/189	0.30	
	rs352140	AA/GA/GG	83/235/226	64/234/200	0.36	
	rs352162	GG/GA/AA	90/242/216	69/236/191	0.41	
TREX1	rs2242150	AA/CA/CC	65/249/222	62/208/227	0.31	
	rs3135941	CC/CT/TT	0/34/488	2/35/460	0.46	
ETS1	rs6590330	AA/GA/GG	100/265/181	36/236/227	$\mathbf{1.6 \times 10^{-8}}$	$\mathbf{2.9 \times 10^{-7}}$
	rs4937333	TT/CT/CC	122/275/142	54/255/188	$\mathbf{9.6 \times 10^{-8}}$	$\mathbf{1.7 \times 10^{-6}}$
TNIP1	rs13168551	TT/CT/CC	20/155/266	27/194/274	0.30	
	rs7708392	GG/GC/CC	20/148/273	29/195/265	0.06	
	rs10036748	CC/CT/TT	21/146/261	27/192/263	0.15	

† *P* values were calculated by Fisher’s Exact test and then were corrected by the Bonferroni criterion.

Table S2. Power analysis of the 18 SNPs in this study.

Gene and SNP	Minor allele	Minor allele frequency	OR	Power (%) # (*P* = 0.05)	Expected sample size *	
					Case	Control
FCGR3A :						
rs11581823	C	0.090	0.84	25	2563	2563
rs7539036	T	0.064	0.8	29	2071	2071
FCGR2A :						
rs4656308	C	0.153	1.12	18	3948	3948
rs1801274	G	0.349	1.08	15	5029	5029
TNFAIP3 :						
rs5029924	T	0.065	1.8	98	250	250
rs5029937	T	0.064	1.88	99	215	215
rs2230926	G	0.064	1.88	99	215	215
rs610604	G	0.099	1	&		
TLR9 :						
rs187084	C	0.366	1.02	&		
rs352140	A	0.375	1.02	&		
rs352162	G	0.381	1.04	&		
TREX1 :						
rs2242150	A	0.344	1.09	17	4031	4031
rs3135941	C	0.036	0.82	16	4523	4523
ETS1 :						
rs6590330	A	0.370	1.66	100	109	109
rs4937333	T	0.426	1.61	100	122	122
TNIP1 :						
rs13168551	T	0.237	0.85	41	1379	1379
rs7708392	G	0.237	0.78	76	575	575
rs10036748	C	0.238	0.82	57	906	906

[#] We selected multiplicative model for the power calculation using the CaTs program (ref.1) and assumed that the prevelance of SLE in our population is 0.06% according to the literature (ref.2 and 3).

* It means that the minimum sample size permits us to detect significant level 0.05 under the detetected effect size with the power of 80%.

[&] Since the effect size is too small, we infer that the minor allele almost does not contribute to SLE and did not perform the power analysis.

Ref.1 Skol AD, Scott LJ, Abecasis GR, Boehnke M: **Joint analysis is more efficient than replication-based analysis for two-stage genome-wide association studies**. *Nat Genet* 2006, **38**:209-213.

Ref.2 Osio-Salido E, Manapat-Reyes H: **Epidemiology of systemic lupus erythematosus in Asia**. *Lupus* 2010, **19**:1365-1373.

Ref.3 Yang W, Zhao M, Hirankarn N, Lau CS, Mok CC, Chan TM, Wong RW, Lee KW, Mok MY, Wong SN, Avihingsanon Y, Lin IO, Lee TL, Ho MH, Lee PP, Wong WH, Sham PC, Lau YL: **ITGAM is associated with disease susceptibility and renal nephritis of systemic lupus erythematosus in Hong Kong Chinese and Thai**. *Hum Mol Genet* 2009, **18**:2063-2070.

Table S3. Allele frequency distribution of the 18 SNPs in controls and different subphenotypes.

Gene	SNP-ID	Minor allele	Major allele	Oneset age (<27, 84)	Control (504)	P†	OR	L95	U95	Malar rash (+, 328)	Control (504)	P	OR	L95	U95	Discoid rash (+, 73)	Control (504)	P	OR	L95	U95	Photosensitivity (+, 78)	Control (504)	P	OR	L95	U95
FCGR3A	rs7539036	T	C	0.061	0.071	0.742	0.844	0.426	1.672	0.057	0.071	0.264	0.785	0.521	1.183	0.089	0.071	0.496	1.271	0.685	2.359	0.058	0.071	0.615	0.796	0.389	1.627
	rs11581823	C	A	0.085	0.097	0.774	0.864	0.480	1.556	0.082	0.097	0.331	0.830	0.583	1.182	0.069	0.097	0.357	0.691	0.351	1.360	0.065	0.097	0.232	0.643	0.327	1.264
FCGR2A	rs4656308	C	T	0.169	0.146	0.48	1.191	0.765	1.854	0.152	0.146	0.723	1.054	0.799	1.391	0.137	0.146	0.9	0.932	0.563	1.541	0.110	0.146	0.265	0.728	0.427	1.242
	rs1801274	G	A	0.374	0.340	0.428	1.157	0.823	1.627	0.353	0.340	0.596	1.061	0.862	1.306	0.336	0.340	1	0.980	0.679	1.416	0.370	0.340	0.467	1.140	0.802	1.622
TNFAIP3	rs5029924	T	C	0.089	0.047	0.038	1.989	1.082	3.657	0.084	0.047	0.003	1.869	1.244	2.809	0.056	0.047	0.675	1.193	0.551	2.586	0.039	0.047	0.836	0.823	0.345	1.962
	rs5029937	T	G	0.090	0.045	0.021	2.117	1.151	3.892	0.084	0.045	0.001	1.944	1.292	2.925	0.055	0.045	0.531	1.235	0.570	2.676	0.039	0.045	1	0.864	0.362	2.061
	rs2230926	G	T	0.089	0.045	0.022	2.089	1.137	3.840	0.084	0.045	0.001	1.957	1.303	2.940	0.055	0.045	0.531	1.235	0.570	2.676	0.038	0.045	0.836	0.852	0.358	2.033
	rs610604	G	T	0.104	0.098	0.78	1.060	0.615	1.825	0.097	0.098	0.933	0.980	0.702	1.368	0.082	0.098	0.652	0.821	0.439	1.535	0.115	0.098	0.477	1.195	0.701	2.038
TLR9	rs352162	G	A	0.375	0.377	1	0.991	0.707	1.390	0.378	0.377	1	1.004	0.820	1.231	0.384	0.377	0.927	1.028	0.719	1.470	0.359	0.377	0.722	0.925	0.651	1.315
	rs352140	A	G	0.349	0.364	0.794	0.941	0.667	1.327	0.364	0.364	1	1.000	0.815	1.228	0.363	0.364	1	0.998	0.696	1.432	0.351	0.364	0.788	0.946	0.663	1.349
	rs187084	C	T	0.369	0.372	1	0.987	0.703	1.385	0.373	0.372	1	1.002	0.816	1.231	0.377	0.372	0.927	1.020	0.712	1.460	0.359	0.372	0.789	0.945	0.664	1.343
TREX1	rs2242150	A	C	0.337	0.334	0.929	1.015	0.717	1.437	0.372	0.334	0.124	1.179	0.958	1.450	0.370	0.334	0.4	1.170	0.816	1.679	0.377	0.334	0.315	1.205	0.848	1.712
	rs3135941	C	T	0.019	0.039	0.258	0.462	0.141	1.513	0.035	0.039	0.69	0.880	0.517	1.499	0.021	0.039	0.35	0.514	0.157	1.684	0.007	0.039	0.053	0.162	0.022	1.189
ETS1	rs6590330	A	G	0.470	0.309	6E-05	1.989	1.428	2.770	0.431	0.309	5E-07	1.694	1.379	2.080	0.375	0.309	0.125	1.344	0.935	1.933	0.435	0.309	0.002	1.725	1.221	2.438
	rs4937333	T	C	0.512	0.365	4E-04	1.824	1.311	2.539	0.489	0.365	8E-07	1.665	1.361	2.035	0.452	0.365	0.045	1.434	1.010	2.036	0.532	0.365	9E-05	1.976	1.407	2.777
TNIP1	rs13168551	T	C	0.167	0.251	0.033	0.598	0.374	0.958	0.214	0.251	0.113	0.813	0.631	1.048	0.246	0.251	1	0.976	0.631	1.509	0.234	0.251	0.742	0.913	0.588	1.418
	rs7708392	G	C	0.162	0.259	0.014	0.553	0.343	0.892	0.209	0.259	0.031	0.756	0.586	0.976	0.238	0.259	0.661	0.894	0.575	1.388	0.216	0.259	0.367	0.787	0.494	1.253
	rs10036748	C	T	0.148	0.255	0.008	0.509	0.306	0.846	0.218	0.255	0.126	0.813	0.630	1.049	0.242	0.255	0.824	0.930	0.598	1.448	0.233	0.255	0.652	0.885	0.562	1.395

Gene	SNP-ID	Oral ulcers (+, 77)	Control (504)	P	OR	L95	U95	Arthritis (+, 341)	Control (504)	P	OR	L95	U95	Serositis (+, 152)	Control (504)	P	OR	L95	U95	Renal disorder (+, 273)	Control (504)	P	OR	L95	U95
FCGR3A	rs7539036	0.058	0.071	0.733	0.807	0.395	1.650	0.062	0.071	0.489	0.864	0.582	1.282	0.043	0.071	0.084	0.585	0.319	1.072	0.057	0.071	0.335	0.792	0.512	1.224
	rs11581823	0.095	0.097	1	0.967	0.536	1.746	0.080	0.097	0.252	0.801	0.563	1.139	0.095	0.097	1	0.975	0.625	1.520	0.075	0.097	0.156	0.747	0.507	1.099
FCGR2A	rs4656308	0.145	0.146	1	0.993	0.612	1.613	0.157	0.146	0.53	1.095	0.834	1.438	0.173	0.146	0.271	1.231	0.870	1.741	0.161	0.146	0.413	1.127	0.844	1.505
	rs1801274	0.380	0.340	0.357	1.190	0.834	1.696	0.351	0.340	0.674	1.048	0.853	1.288	0.282	0.340	0.067	0.762	0.573	1.012	0.350	0.340	0.694	1.045	0.839	1.302
TNFAIP3	rs5029924	0.053	0.047	0.683	1.143	0.528	2.475	0.082	0.047	0.005	1.803	1.200	2.708	0.083	0.047	0.021	1.831	1.103	3.040	0.068	0.047	0.098	1.481	0.946	2.318
	rs5029937	0.053	0.045	0.676	1.184	0.547	2.563	0.080	0.045	0.003	1.862	1.238	2.801	0.086	0.045	0.009	2.008	1.216	3.313	0.067	0.045	0.072	1.528	0.973	2.400
	rs2230926	0.052	0.045	0.679	1.168	0.540	2.527	0.081	0.045	0.003	1.869	1.245	2.807	0.086	0.045	0.009	1.993	1.208	3.289	0.068	0.045	0.057	1.555	0.994	2.434
	rs610604	0.092	0.098	0.884	0.930	0.516	1.674	0.090	0.098	0.611	0.906	0.648	1.267	0.093	0.098	0.825	0.936	0.602	1.456	0.092	0.098	0.718	0.931	0.651	1.332
TLR9	rs352162	0.331	0.377	0.284	0.818	0.571	1.172	0.391	0.377	0.573	1.062	0.869	1.297	0.378	0.377	1	1.005	0.771	1.310	0.405	0.377	0.299	1.124	0.907	1.391
	rs352140	0.309	0.364	0.204	0.784	0.543	1.132	0.377	0.364	0.57	1.061	0.867	1.298	0.361	0.364	0.946	0.989	0.757	1.293	0.386	0.364	0.408	1.099	0.886	1.364
	rs187084	0.329	0.372	0.321	0.827	0.576	1.188	0.382	0.372	0.679	1.045	0.853	1.280	0.372	0.372	1	0.998	0.765	1.303	0.397	0.372	0.349	1.109	0.894	1.376
TREX1	rs2242150	0.380	0.334	0.269	1.222	0.857	1.743	0.367	0.334	0.172	1.157	0.942	1.420	0.362	0.334	0.366	1.133	0.865	1.485	0.383	0.334	0.057	1.239	0.997	1.541
	rs3135941	0.035	0.039	1	0.881	0.341	2.273	0.029	0.039	0.339	0.733	0.420	1.279	0.031	0.039	0.6	0.779	0.373	1.627	0.027	0.039	0.24	0.670	0.360	1.245
ETS1	rs6590330	0.331	0.309	0.576	1.109	0.773	1.592	0.415	0.309	9E-06	1.592	1.299	1.952	0.417	0.309	6E-04	1.600	1.227	2.088	0.424	0.309	7E-06	1.650	1.328	2.050
	rs4937333	0.435	0.365	0.107	1.339	0.949	1.888	0.479	0.365	5E-06	1.599	1.310	1.951	0.463	0.365	0.003	1.498	1.152	1.947	0.485	0.365	6E-06	1.638	1.323	2.026
TNIP1	rs13168551	0.237	0.251	0.822	0.931	0.595	1.457	0.229	0.251	0.35	0.887	0.693	1.136	0.271	0.251	0.507	1.113	0.808	1.535	0.211	0.251	0.12	0.798	0.608	1.048
	rs7708392	0.233	0.259	0.581	0.872	0.558	1.363	0.229	0.259	0.214	0.849	0.664	1.087	0.259	0.259	1	1.000	0.721	1.386	0.208	0.259	0.043	0.752	0.572	0.989
	rs10036748	0.232	0.255	0.647	0.882	0.556	1.400	0.227	0.255	0.256	0.858	0.668	1.102	0.263	0.255	0.801	1.042	0.751	1.448	0.217	0.255	0.149	0.809	0.614	1.066

Gene	SNP-ID	Neurologic disorder (+, 50)	Control (504)	P	OR	L95	U95	Hematologic disorder (-, 370)	Control (504)	P	OR	L95	U95	Immunologic disorder (+, 340)	Control (504)	P	OR	L95	U95	Anti-nuclear antibody (+, 437)	Control (504)	P	OR	L95	U95
FCGR3A	rs7539036	0.031	0.071	0.143	0.411	0.127	1.329	0.059	0.071	0.327	0.809	0.547	1.196	0.065	0.071	0.694	0.911	0.617	1.345	0.059	0.071	0.302	0.814	0.561	1.180
	rs11581823	0.063	0.097	0.358	0.617	0.263	1.449	0.076	0.097	0.14	0.761	0.537	1.078	0.074	0.097	0.108	0.735	0.512	1.055	0.079	0.097	0.187	0.799	0.576	1.108
FCGR2A	rs4656308	0.153	0.146	0.881	1.061	0.595	1.889	0.148	0.146	0.945	1.016	0.776	1.330	0.144	0.146	1	0.990	0.750	1.307	0.155	0.146	0.603	1.074	0.833	1.386
	rs1801274	0.388	0.340	0.373	1.229	0.802	1.884	0.368	0.340	0.24	1.131	0.926	1.381	0.368	0.340	0.249	1.131	0.922	1.389	0.362	0.340	0.33	1.101	0.910	1.333
TNFAIP3	rs5029924	0.130	0.047	0.002	3.032	1.575	5.837	0.079	0.047	0.007	1.746	1.168	2.610	0.078	0.047	0.011	1.726	1.145	2.601	0.076	0.047	0.011	1.670	1.130	2.468
	rs5029937	0.130	0.045	0.001	3.184	1.654	6.130	0.079	0.045	0.004	1.839	1.231	2.749	0.079	0.045	0.006	1.819	1.207	2.740	0.075	0.045	0.006	1.734	1.172	2.564
	rs2230926	0.130	0.045	0.001	3.184	1.654	6.130	0.080	0.045	0.003	1.852	1.241	2.763	0.080	0.045	0.004	1.844	1.226	2.774	0.076	0.045	0.006	1.745	1.181	2.578
	rs610604	0.080	0.098	0.722	0.797	0.376	1.690	0.091	0.098	0.679	0.920	0.664	1.276	0.092	0.098	0.673	0.928	0.665	1.297	0.094	0.098	0.814	0.956	0.702	1.302
TLR9	rs352162	0.400	0.377	0.666	1.102	0.724	1.677	0.359	0.377	0.45	0.926	0.760	1.128	0.397	0.377	0.443	1.087	0.889	1.328	0.378	0.377	0.962	1.006	0.834	1.214
	rs352140	0.380	0.364	0.745	1.073	0.703	1.640	0.347	0.364	0.509	0.932	0.764	1.138	0.378	0.364	0.536	1.066	0.871	1.305	0.363	0.364	1	0.998	0.826	1.205
	rs187084	0.400	0.372	0.589	1.125	0.738	1.713	0.352	0.372	0.415	0.917	0.750	1.120	0.390	0.372	0.47	1.080	0.882	1.321	0.369	0.372	0.923	0.987	0.817	1.194
TREX1	rs2242150	0.347	0.334	0.823	1.059	0.685	1.639	0.350	0.334	0.504	1.075	0.879	1.315	0.357	0.334	0.344	1.106	0.900	1.358	0.353	0.334	0.405	1.086	0.897	1.316
	rs3135941	0.000	0.039	0.042	0.000	0.000	nan	0.029	0.039	0.29	0.742	0.433	1.273	0.023	0.039	0.089	0.573	0.313	1.048	0.028	0.039	0.247	0.715	0.426	1.199
ETS1	rs6590330	0.398	0.309	0.087	1.481	0.967	2.268	0.431	0.309	2E-07	1.694	1.389	2.065	0.435	0.309	2E-07	1.721	1.405	2.109	0.424	0.309	3E-07	1.648	1.362	1.993
	rs4937333	0.429	0.365	0.229	1.304	0.856	1.985	0.482	0.365	1E-06	1.618	1.332	1.966	0.494	0.365	2E-07	1.697	1.390	2.072	0.478	0.365	1E-06	1.591	1.320	1.916
TNIP1	rs13168551	0.216	0.251	0.521	0.824	0.486	1.397	0.225	0.251	0.274	0.871	0.684	1.108	0.216	0.251	0.135	0.826	0.644	1.059	0.211	0.251	0.062	0.798	0.633	1.007
	rs7708392	0.216	0.259	0.444	0.789	0.466	1.337	0.222	0.259	0.114	0.817	0.642	1.040	0.210	0.259	0.034	0.761	0.592	0.978	0.208	0.259	0.017	0.750	0.595	0.947
	rs10036748	0.226	0.255	0.603	0.853	0.502	1.451	0.231	0.255	0.297	0.875	0.686	1.116	0.218	0.255	0.115	0.814	0.633	1.047	0.215	0.255	0.06	0.797	0.631	1.007

Gene	SNP-ID	Minor allele	Major allele	Oneset age (>= 27, 366)	Control (504)	P	OR	L95	U95	Malar rash (-, 122)	Control (504)	P	OR	L95	U95	Discoid rash (-, 377)	Control (504)	P	OR	L95	U95	Photosensitivity (-, 372)	Control (504)	P	OR	L95	U95
FCGR3A	rs7539036	T	C	0.059	0.071	0.328	0.816	0.552	1.207	0.066	0.071	0.889	0.920	0.525	1.614	0.054	0.071	0.138	0.737	0.494	1.098	0.060	0.071	0.38	0.827	0.560	1.219
	rs11581823	C	A	0.078	0.097	0.193	0.783	0.554	1.108	0.071	0.097	0.26	0.712	0.416	1.219	0.081	0.097	0.267	0.819	0.583	1.151	0.082	0.097	0.305	0.832	0.592	1.168
FCGR2A	rs4656308	C	T	0.150	0.146	0.837	1.037	0.792	1.357	0.157	0.146	0.686	1.093	0.741	1.612	0.157	0.146	0.542	1.092	0.838	1.422	0.163	0.146	0.345	1.140	0.876	1.483
	rs1801274	G	A	0.357	0.340	0.472	1.079	0.883	1.319	0.379	0.340	0.258	1.185	0.885	1.587	0.365	0.340	0.286	1.117	0.915	1.362	0.358	0.340	0.444	1.084	0.887	1.324
TNFAIP3	rs5029924	T	C	0.073	0.047	0.027	1.603	1.064	2.414	0.054	0.047	0.616	1.162	0.616	2.190	0.080	0.047	0.006	1.769	1.187	2.637	0.084	0.047	0.002	1.861	1.252	2.766
	rs5029937	T	G	0.072	0.045	0.019	1.649	1.093	2.487	0.053	0.045	0.611	1.199	0.636	2.260	0.079	0.045	0.003	1.836	1.230	2.739	0.083	0.045	0.002	1.926	1.294	2.866
	rs2230926	G	T	0.073	0.045	0.015	1.668	1.108	2.512	0.053	0.045	0.611	1.199	0.636	2.260	0.080	0.045	0.003	1.848	1.240	2.753	0.084	0.045	0.001	1.943	1.307	2.888
	rs610604	G	T	0.092	0.098	0.679	0.926	0.668	1.284	0.087	0.098	0.629	0.871	0.531	1.427	0.096	0.098	0.935	0.976	0.709	1.344	0.089	0.098	0.562	0.900	0.649	1.249
TLR9	rs352162	G	A	0.378	0.377	1	1.005	0.825	1.223	0.376	0.377	1	0.996	0.745	1.331	0.376	0.377	1	0.997	0.820	1.212	0.381	0.377	0.881	1.019	0.837	1.239
	rs352140	A	G	0.365	0.364	0.96	1.008	0.827	1.230	0.360	0.364	0.941	0.983	0.734	1.317	0.362	0.364	1	0.995	0.817	1.212	0.365	0.364	0.96	1.006	0.826	1.226
	rs187084	C	T	0.368	0.372	0.879	0.984	0.806	1.201	0.357	0.372	0.708	0.937	0.698	1.259	0.367	0.372	0.84	0.978	0.802	1.192	0.371	0.372	0.96	0.993	0.814	1.211
TREX1	rs2242150	A	C	0.359	0.334	0.304	1.115	0.912	1.364	0.310	0.334	0.493	0.896	0.662	1.212	0.352	0.334	0.443	1.082	0.886	1.322	0.350	0.334	0.505	1.074	0.879	1.313
	rs3135941	C	T	0.031	0.039	0.428	0.783	0.460	1.333	0.013	0.039	0.045	0.313	0.096	1.020	0.030	0.039	0.357	0.765	0.450	1.302	0.033	0.039	0.603	0.844	0.503	1.417
ETS1	rs6590330	A	G	0.410	0.309	1E-05	1.558	1.276	1.903	0.398	0.309	0.01	1.478	1.107	1.974	0.431	0.309	2E-07	1.693	1.390	2.063	0.419	0.309	2E-06	1.614	1.323	1.968
	rs4937333	T	C	0.468	0.365	2E-05	1.528	1.257	1.858	0.441	0.365	0.037	1.370	1.027	1.826	0.481	0.365	2E-06	1.611	1.327	1.955	0.464	0.365	5E-05	1.506	1.239	1.830
TNIP1	rs13168551	T	C	0.231	0.251	0.394	0.899	0.707	1.144	0.232	0.251	0.649	0.904	0.629	1.299	0.213	0.251	0.099	0.810	0.635	1.033	0.216	0.251	0.127	0.822	0.645	1.048
	rs7708392	G	C	0.228	0.259	0.181	0.844	0.663	1.075	0.232	0.259	0.471	0.866	0.602	1.244	0.210	0.259	0.033	0.764	0.599	0.975	0.215	0.259	0.053	0.785	0.616	0.999
	rs10036748	C	T	0.239	0.255	0.502	0.915	0.719	1.164	0.234	0.255	0.579	0.890	0.614	1.290	0.218	0.255	0.11	0.814	0.637	1.039	0.220	0.255	0.126	0.823	0.645	1.050

Gene	SNP-ID	Oral ulcers (-, 373)	Control (504)	P	OR	L95	U95	Arthritis (-, 109)	Control (504)	P	OR	L95	U95	Serositis (-, 298)	Control (504)	P	OR	L95	U95	Renal disorder (-, 177)	Control (504)	P	OR	L95	U95
FCGR3A	rs7539036	0.060	0.071	0.38	0.824	0.559	1.216	0.050	0.071	0.3	0.691	0.360	1.327	0.068	0.071	0.839	0.946	0.633	1.413	0.063	0.071	0.626	0.867	0.529	1.421
	rs11581823	0.076	0.097	0.141	0.765	0.541	1.081	0.079	0.097	0.441	0.791	0.461	1.356	0.071	0.097	0.095	0.712	0.487	1.041	0.087	0.097	0.594	0.879	0.571	1.353
FCGR2A	rs4656308	0.155	0.146	0.587	1.080	0.828	1.408	0.142	0.146	1	0.973	0.640	1.479	0.144	0.146	0.941	0.984	0.737	1.314	0.142	0.146	0.93	0.972	0.687	1.375
	rs1801274	0.356	0.340	0.507	1.075	0.880	1.312	0.390	0.340	0.182	1.240	0.917	1.678	0.400	0.340	0.017	1.294	1.048	1.597	0.376	0.340	0.24	1.172	0.909	1.510
TNFAIP3	rs5029924	0.081	0.047	0.004	1.785	1.198	2.660	0.060	0.047	0.487	1.287	0.682	2.429	0.073	0.047	0.041	1.595	1.036	2.455	0.089	0.047	0.007	1.984	1.234	3.190
	rs5029937	0.080	0.045	0.003	1.852	1.241	2.763	0.060	0.045	0.378	1.351	0.716	2.551	0.070	0.045	0.039	1.597	1.033	2.470	0.088	0.045	0.004	2.058	1.280	3.308
	rs2230926	0.081	0.045	0.002	1.869	1.255	2.785	0.060	0.045	0.377	1.365	0.723	2.576	0.071	0.045	0.03	1.621	1.051	2.501	0.088	0.045	0.004	2.045	1.273	3.287
	rs610604	0.094	0.098	0.806	0.955	0.691	1.318	0.107	0.098	0.708	1.092	0.676	1.765	0.095	0.098	0.861	0.957	0.678	1.352	0.097	0.098	1	0.980	0.650	1.477
TLR9	rs352162	0.387	0.377	0.69	1.044	0.858	1.269	0.335	0.377	0.278	0.832	0.611	1.133	0.377	0.377	1	1.000	0.811	1.234	0.335	0.377	0.176	0.833	0.645	1.076
	rs352140	0.373	0.364	0.688	1.043	0.857	1.270	0.317	0.364	0.211	0.811	0.593	1.109	0.363	0.364	1	0.999	0.808	1.234	0.327	0.364	0.219	0.850	0.657	1.099
	rs187084	0.377	0.372	0.88	1.020	0.837	1.242	0.326	0.372	0.213	0.815	0.597	1.113	0.367	0.372	0.871	0.978	0.790	1.209	0.325	0.372	0.119	0.811	0.626	1.051
TREX1	rs2242150	0.350	0.334	0.506	1.072	0.877	1.310	0.317	0.334	0.635	0.923	0.674	1.264	0.351	0.334	0.511	1.078	0.870	1.335	0.310	0.334	0.427	0.897	0.690	1.167
	rs3135941	0.027	0.039	0.227	0.692	0.400	1.196	0.028	0.039	0.553	0.693	0.290	1.658	0.028	0.039	0.256	0.695	0.385	1.255	0.032	0.039	0.623	0.804	0.407	1.588
ETS1	rs6590330	0.440	0.309	2E-08	1.763	1.446	2.149	0.440	0.309	3E-04	1.763	1.307	2.378	0.424	0.309	4E-06	1.649	1.335	2.037	0.418	0.309	2E-04	1.606	1.250	2.064
	rs4937333	0.485	0.365	7E-07	1.636	1.347	1.987	0.467	0.365	0.006	1.523	1.129	2.054	0.483	0.365	5E-06	1.624	1.320	1.997	0.462	0.365	0.002	1.495	1.167	1.916
TNIP1	rs13168551	0.215	0.251	0.114	0.819	0.643	1.044	0.189	0.251	0.088	0.697	0.467	1.039	0.193	0.251	0.015	0.715	0.547	0.935	0.231	0.251	0.533	0.898	0.658	1.224
	rs7708392	0.211	0.259	0.034	0.768	0.602	0.980	0.174	0.259	0.018	0.604	0.400	0.913	0.194	0.259	0.006	0.691	0.529	0.902	0.226	0.259	0.279	0.835	0.612	1.140
	rs10036748	0.220	0.255	0.126	0.824	0.646	1.051	0.207	0.255	0.183	0.761	0.513	1.129	0.202	0.255	0.029	0.739	0.566	0.966	0.229	0.255	0.394	0.868	0.636	1.184

Gene	SNP-ID	Neurologic disorder (-, 400)	Control (504)	P	OR	L95	U95	Hematologic disorder (-, 80)	Control (504)	P	OR	L95	U95	Immunologic disorder (-, 110)	Control (504)	P	OR	L95	U95	Anti-nuclear antibody (-, 13)	Control (504)	P	OR	L95	U95
FCGR3A	rs7539036	0.063	0.071	0.508	0.874	0.601	1.271	0.063	0.071	0.867	0.878	0.443	1.741	0.041	0.071	0.132	0.555	0.273	1.127	0.077	0.071	0.709	1.083	0.251	4.677
	rs11581823	0.081	0.097	0.275	0.821	0.588	1.145	0.095	0.097	1	0.971	0.547	1.723	0.097	0.097	1	0.997	0.606	1.641	0.077	0.097	1	0.772	0.180	3.316
FCGR2A	rs4656308	0.154	0.146	0.641	1.065	0.821	1.383	0.181	0.146	0.236	1.299	0.838	2.015	0.182	0.146	0.178	1.304	0.887	1.917	0.115	0.146	1	0.766	0.227	2.582
	rs1801274	0.357	0.340	0.484	1.077	0.886	1.311	0.325	0.340	0.787	0.935	0.655	1.334	0.336	0.340	0.938	0.984	0.722	1.340	0.308	0.340	0.836	0.863	0.371	2.004
TNFAIP3	rs5029924	0.069	0.047	0.049	1.514	1.009	2.272	0.063	0.047	0.428	1.353	0.667	2.742	0.069	0.047	0.174	1.514	0.828	2.770	0.083	0.047	0.321	1.844	0.421	8.088
	rs5029937	0.068	0.045	0.037	1.564	1.041	2.349	0.056	0.045	0.542	1.270	0.609	2.652	0.065	0.045	0.221	1.477	0.796	2.742	0.077	0.045	0.335	1.776	0.407	7.748
	rs2230926	0.069	0.045	0.03	1.578	1.052	2.366	0.056	0.045	0.542	1.270	0.609	2.652	0.064	0.045	0.227	1.448	0.780	2.688	0.077	0.045	0.335	1.776	0.407	7.748
	rs610604	0.096	0.098	0.873	0.970	0.708	1.329	0.106	0.098	0.776	1.089	0.632	1.877	0.100	0.098	0.901	1.018	0.625	1.657	0.077	0.098	1	0.764	0.178	3.280
TLR9	rs352162	0.375	0.377	0.922	0.990	0.817	1.200	0.463	0.377	0.044	1.422	1.016	1.990	0.318	0.377	0.105	0.771	0.565	1.053	0.346	0.377	0.839	0.875	0.386	1.982
	rs352140	0.360	0.364	0.921	0.986	0.812	1.197	0.431	0.364	0.113	1.328	0.947	1.863	0.314	0.364	0.186	0.800	0.585	1.094	0.346	0.364	1	0.927	0.409	2.101
	rs187084	0.365	0.372	0.766	0.968	0.797	1.176	0.444	0.372	0.095	1.346	0.960	1.887	0.301	0.372	0.05	0.726	0.528	0.999	0.346	0.372	0.84	0.893	0.394	2.025
TREX1	rs2242150	0.356	0.334	0.341	1.101	0.905	1.340	0.375	0.334	0.323	1.196	0.846	1.691	0.349	0.334	0.693	1.067	0.784	1.452	0.423	0.334	0.401	1.462	0.664	3.219
	rs3135941	0.032	0.039	0.521	0.817	0.490	1.363	0.025	0.039	0.502	0.636	0.224	1.805	0.046	0.039	0.573	1.189	0.584	2.420	0.038	0.039	1	0.980	0.129	7.415
ETS1	rs6590330	0.424	0.309	4E-07	1.652	1.360	2.007	0.380	0.309	0.081	1.372	0.968	1.943	0.382	0.309	0.038	1.384	1.022	1.874	0.346	0.309	0.672	1.186	0.523	2.690
	rs4937333	0.482	0.365	8E-07	1.618	1.338	1.958	0.448	0.365	0.05	1.411	1.002	1.988	0.422	0.365	0.123	1.269	0.942	1.710	0.423	0.365	0.543	1.275	0.579	2.805
TNIP1	rs13168551	0.219	0.251	0.152	0.839	0.662	1.064	0.188	0.251	0.126	0.690	0.433	1.101	0.226	0.251	0.562	0.875	0.593	1.290	0.500	0.251	0.018	2.992	1.231	7.273
	rs7708392	0.215	0.259	0.049	0.785	0.618	0.996	0.185	0.259	0.068	0.649	0.407	1.034	0.232	0.259	0.503	0.866	0.589	1.274	0.455	0.259	0.049	2.388	1.019	5.594
	rs10036748	0.222	0.255	0.132	0.830	0.654	1.055	0.185	0.255	0.084	0.661	0.415	1.053	0.235	0.255	0.629	0.896	0.609	1.320	0.455	0.255	0.047	2.432	1.038	5.700

Gene	SNP-ID	Minor allele	Major allele	Oneset age (<27, 84)	Oneset age (>= 27, 366)	P	OR	L95	U95	Malar rash (+, 328)	Malar rash (-, 122)	P	OR	L95	U95	Discoid rash (+, 73)	Discoid rash (-, 377)	P	OR	L95	U95	Photosensitivity (+, 78)	Photosensitivity (-, 372)	P	OR	L95	U95
FCGR3A	rs7539036	T	C	0.061	0.059	0.857	1.034	0.509	2.104	0.057	0.066	0.633	0.853	0.465	1.563	0.089	0.054	0.123	1.725	0.898	3.313	0.058	0.060	1	0.963	0.460	2.016
	rs11581823	C	A	0.085	0.078	0.749	1.103	0.598	2.034	0.082	0.071	0.675	1.166	0.661	2.057	0.069	0.081	0.737	0.843	0.421	1.689	0.065	0.082	0.622	0.773	0.387	1.546
FCGR2A	rs4656308	C	T	0.169	0.150	0.552	1.149	0.729	1.809	0.152	0.157	0.917	0.965	0.642	1.449	0.137	0.157	0.616	0.853	0.512	1.423	0.110	0.163	0.111	0.639	0.372	1.097
	rs1801274	G	A	0.374	0.357	0.72	1.072	0.756	1.521	0.353	0.379	0.48	0.895	0.659	1.216	0.336	0.365	0.511	0.878	0.604	1.277	0.370	0.358	0.783	1.052	0.734	1.509
TNFAIP3	rs5029924	T	C	0.089	0.073	0.518	1.241	0.682	2.261	0.084	0.054	0.155	1.609	0.862	3.001	0.056	0.080	0.391	0.675	0.315	1.443	0.039	0.084	0.065	0.442	0.188	1.041
	rs5029937	T	G	0.090	0.072	0.416	1.284	0.704	2.341	0.084	0.053	0.154	1.621	0.868	3.026	0.055	0.079	0.391	0.673	0.315	1.440	0.039	0.083	0.065	0.449	0.190	1.057
	rs2230926	G	T	0.089	0.073	0.517	1.252	0.688	2.281	0.084	0.053	0.155	1.632	0.875	3.043	0.055	0.080	0.392	0.669	0.313	1.430	0.038	0.084	0.065	0.439	0.186	1.033
	rs610604	G	T	0.104	0.092	0.657	1.144	0.653	2.006	0.097	0.087	0.701	1.126	0.671	1.889	0.082	0.096	0.756	0.841	0.444	1.592	0.115	0.089	0.295	1.328	0.764	2.307
TLR9	rs352162	G	A	0.375	0.378	1	0.987	0.698	1.395	0.378	0.376	1	1.009	0.744	1.367	0.384	0.376	0.926	1.031	0.716	1.485	0.359	0.381	0.65	0.908	0.634	1.301
	rs352140	A	G	0.349	0.365	0.721	0.933	0.655	1.328	0.364	0.360	0.938	1.017	0.748	1.383	0.363	0.362	1	1.003	0.694	1.450	0.351	0.365	0.783	0.940	0.654	1.352
	rs187084	C	T	0.369	0.368	1	1.003	0.708	1.420	0.373	0.357	0.695	1.069	0.785	1.457	0.377	0.367	0.851	1.043	0.723	1.504	0.359	0.371	0.855	0.951	0.664	1.363
TREX1	rs2242150	A	C	0.337	0.359	0.653	0.910	0.638	1.299	0.372	0.310	0.098	1.316	0.960	1.805	0.370	0.352	0.705	1.082	0.749	1.563	0.377	0.350	0.578	1.121	0.783	1.606
	rs3135941	C	T	0.019	0.031	0.601	0.590	0.175	1.996	0.035	0.013	0.109	2.816	0.835	9.496	0.021	0.030	0.785	0.671	0.198	2.273	0.007	0.033	0.104	0.192	0.026	1.431
ETS1	rs6590330	A	G	0.470	0.410	0.166	1.276	0.911	1.788	0.431	0.398	0.403	1.146	0.849	1.546	0.375	0.431	0.232	0.794	0.550	1.146	0.435	0.419	0.72	1.069	0.753	1.518
	rs4937333	T	C	0.512	0.468	0.343	1.193	0.851	1.673	0.489	0.441	0.223	1.215	0.901	1.640	0.452	0.481	0.527	0.890	0.623	1.272	0.532	0.464	0.133	1.312	0.928	1.856
TNIP1	rs13168551	T	C	0.167	0.231	0.109	0.666	0.409	1.084	0.214	0.232	0.612	0.900	0.607	1.334	0.246	0.213	0.471	1.204	0.763	1.901	0.234	0.216	0.635	1.111	0.702	1.760
	rs7708392	G	C	0.162	0.228	0.105	0.655	0.399	1.076	0.209	0.232	0.539	0.874	0.589	1.297	0.238	0.210	0.545	1.170	0.738	1.856	0.216	0.215	1	1.003	0.619	1.627
	rs10036748	C	T	0.148	0.239	0.026	0.556	0.329	0.939	0.218	0.234	0.68	0.914	0.612	1.364	0.242	0.218	0.549	1.143	0.720	1.815	0.233	0.220	0.807	1.076	0.670	1.727

Gene	SNP-ID	Oral ulcers (+, 77)	Oral ulcers (-, 373)	P	OR	L95	U95	Arthritis (+, 341)	Arthritis (-, 109)	P	OR	L95	U95	Serositis (+, 152)	Serositis (-, 298)	P	OR	L95	U95	Renal disorder (+, 273)	Renal disorder (-, 177)	P	OR	L95	U95
FCGR3A	rs7539036	0.058	0.060	1	0.979	0.468	2.050	0.062	0.050	0.622	1.251	0.632	2.474	0.043	0.068	0.177	0.619	0.326	1.175	0.057	0.063	0.773	0.914	0.520	1.605
	rs11581823	0.095	0.076	0.504	1.265	0.684	2.338	0.080	0.079	1	1.012	0.573	1.788	0.095	0.071	0.235	1.368	0.830	2.256	0.075	0.087	0.526	0.850	0.518	1.392
FCGR2A	rs4656308	0.145	0.155	0.806	0.920	0.561	1.507	0.157	0.142	0.666	1.126	0.730	1.736	0.173	0.144	0.28	1.251	0.858	1.823	0.161	0.142	0.449	1.160	0.796	1.691
	rs1801274	0.380	0.356	0.577	1.107	0.770	1.591	0.351	0.390	0.33	0.845	0.617	1.158	0.282	0.400	5E-04	0.589	0.436	0.796	0.350	0.376	0.432	0.892	0.674	1.180
TNFAIP3	rs5029924	0.053	0.081	0.312	0.640	0.300	1.369	0.082	0.060	0.378	1.401	0.750	2.617	0.083	0.073	0.596	1.148	0.687	1.919	0.068	0.089	0.248	0.746	0.454	1.227
	rs5029937	0.053	0.080	0.311	0.639	0.299	1.367	0.080	0.060	0.376	1.378	0.737	2.576	0.086	0.070	0.421	1.257	0.753	2.098	0.067	0.088	0.245	0.743	0.450	1.225
	rs2230926	0.052	0.081	0.246	0.625	0.292	1.334	0.081	0.060	0.377	1.370	0.733	2.559	0.086	0.071	0.427	1.229	0.738	2.047	0.068	0.088	0.303	0.760	0.463	1.250
	rs610604	0.092	0.094	1	0.974	0.533	1.779	0.090	0.107	0.503	0.830	0.500	1.376	0.093	0.095	1	0.978	0.607	1.575	0.092	0.097	0.816	0.951	0.601	1.503
TLR9	rs352162	0.331	0.387	0.202	0.784	0.543	1.131	0.391	0.335	0.149	1.276	0.926	1.759	0.378	0.377	1	1.005	0.756	1.337	0.405	0.335	0.041	1.348	1.020	1.783
	rs352140	0.309	0.373	0.14	0.751	0.517	1.093	0.377	0.317	0.124	1.308	0.945	1.810	0.361	0.363	1	0.990	0.742	1.321	0.386	0.327	0.076	1.293	0.976	1.715
	rs187084	0.329	0.377	0.31	0.811	0.560	1.174	0.382	0.326	0.146	1.282	0.928	1.771	0.372	0.367	0.884	1.021	0.766	1.360	0.397	0.325	0.033	1.367	1.031	1.814
TREX1	rs2242150	0.380	0.350	0.513	1.140	0.793	1.639	0.367	0.317	0.192	1.253	0.905	1.735	0.362	0.351	0.767	1.052	0.787	1.406	0.383	0.310	0.031	1.381	1.038	1.838
	rs3135941	0.035	0.027	0.588	1.273	0.470	3.450	0.029	0.028	1	1.057	0.417	2.682	0.031	0.028	0.831	1.121	0.489	2.568	0.027	0.032	0.682	0.833	0.374	1.856
ETS1	rs6590330	0.331	0.440	0.015	0.629	0.437	0.907	0.415	0.440	0.529	0.903	0.664	1.229	0.417	0.424	0.886	0.970	0.732	1.286	0.424	0.418	0.89	1.027	0.782	1.348
	rs4937333	0.435	0.485	0.287	0.818	0.576	1.161	0.479	0.467	0.813	1.050	0.770	1.431	0.463	0.483	0.617	0.923	0.697	1.221	0.485	0.462	0.535	1.095	0.835	1.436
TNIP1	rs13168551	0.237	0.215	0.626	1.136	0.712	1.811	0.229	0.189	0.298	1.273	0.833	1.945	0.271	0.193	0.021	1.556	1.080	2.243	0.211	0.231	0.52	0.890	0.621	1.274
	rs7708392	0.233	0.211	0.626	1.135	0.712	1.810	0.229	0.174	0.141	1.405	0.908	2.175	0.259	0.194	0.052	1.447	1.000	2.096	0.208	0.226	0.579	0.901	0.627	1.293
	rs10036748	0.232	0.220	0.804	1.071	0.663	1.731	0.227	0.207	0.601	1.126	0.740	1.714	0.263	0.202	0.08	1.410	0.973	2.043	0.217	0.229	0.712	0.933	0.650	1.339

Gene	SNP-ID	Neurologic disorder (+, 50)	Neurologic disorder (-, 400)	P	OR	L95	U95	Hematologic disorder (-, 370)	Hematologic disorder (-, 80)	P	OR	L95	U95	Immunologic disorder (+, 340)	Immunologic disorder (-, 110)	P	OR	L95	U95	Anti-nuclear antibody (+, 437)	Anti-nuclear antibody (-, 13)	P	OR	L95	U95
FCGR3A	rs7539036	0.031	0.063	0.26	0.470	0.144	1.536	0.059	0.063	0.853	0.921	0.453	1.874	0.065	0.041	0.249	1.643	0.789	3.422	0.059	0.077	0.664	0.751	0.173	3.266
	rs11581823	0.063	0.081	0.689	0.752	0.317	1.786	0.076	0.095	0.418	0.784	0.431	1.426	0.074	0.097	0.31	0.737	0.432	1.260	0.079	0.077	1	1.036	0.240	4.475
FCGR2A	rs4656308	0.153	0.154	1	0.996	0.556	1.783	0.148	0.181	0.278	0.782	0.498	1.228	0.144	0.182	0.196	0.759	0.507	1.138	0.155	0.115	0.785	1.403	0.416	4.740
	rs1801274	0.388	0.357	0.578	1.141	0.741	1.757	0.368	0.325	0.318	1.210	0.841	1.740	0.368	0.336	0.419	1.150	0.835	1.585	0.362	0.308	0.681	1.276	0.549	2.969
TNFAIP3	rs5029924	0.130	0.069	0.043	2.002	1.052	3.812	0.079	0.063	0.621	1.291	0.645	2.584	0.078	0.069	0.769	1.140	0.629	2.067	0.076	0.083	0.704	0.905	0.208	3.934
	rs5029937	0.130	0.068	0.041	2.037	1.069	3.882	0.079	0.056	0.408	1.448	0.702	2.987	0.079	0.065	0.556	1.231	0.669	2.266	0.075	0.077	1	0.976	0.226	4.222
	rs2230926	0.130	0.069	0.042	2.019	1.060	3.843	0.080	0.056	0.409	1.458	0.707	3.005	0.080	0.064	0.557	1.273	0.693	2.340	0.076	0.077	1	0.983	0.227	4.249
	rs610604	0.080	0.096	0.718	0.822	0.384	1.757	0.091	0.106	0.551	0.845	0.482	1.482	0.092	0.100	0.692	0.912	0.546	1.522	0.094	0.077	1	1.252	0.291	5.392
TLR9	rs352162	0.400	0.375	0.662	1.113	0.727	1.702	0.359	0.463	0.015	0.651	0.461	0.920	0.397	0.318	0.038	1.409	1.021	1.946	0.378	0.346	0.839	1.150	0.507	2.610
	rs352140	0.380	0.360	0.741	1.089	0.709	1.672	0.347	0.431	0.056	0.702	0.496	0.994	0.378	0.314	0.09	1.332	0.963	1.843	0.363	0.346	1	1.076	0.474	2.442
	rs187084	0.400	0.365	0.51	1.162	0.759	1.778	0.352	0.444	0.037	0.681	0.481	0.964	0.390	0.301	0.019	1.487	1.069	2.066	0.369	0.346	1	1.106	0.487	2.509
TREX1	rs2242150	0.347	0.356	0.911	0.962	0.619	1.495	0.350	0.375	0.584	0.899	0.630	1.281	0.357	0.349	0.871	1.036	0.752	1.427	0.353	0.423	0.533	0.743	0.337	1.638
	rs3135941	0.000	0.032	0.1	0.000	0.000	nan	0.029	0.025	1	1.167	0.395	3.447	0.023	0.046	0.097	0.482	0.213	1.089	0.028	0.038	0.536	0.730	0.095	5.611
ETS1	rs6590330	0.398	0.424	0.665	0.896	0.584	1.376	0.431	0.380	0.25	1.235	0.868	1.757	0.435	0.382	0.182	1.244	0.911	1.699	0.424	0.346	0.547	1.389	0.612	3.151
	rs4937333	0.429	0.482	0.336	0.806	0.527	1.231	0.482	0.448	0.478	1.147	0.809	1.626	0.494	0.422	0.072	1.337	0.982	1.821	0.478	0.423	0.691	1.248	0.567	2.748
TNIP1	rs13168551	0.216	0.219	1	0.982	0.571	1.688	0.225	0.188	0.409	1.261	0.777	2.046	0.216	0.226	0.831	0.945	0.624	1.430	0.211	0.500	0.005	0.267	0.109	0.653
	rs7708392	0.216	0.215	1	1.006	0.585	1.730	0.222	0.185	0.409	1.259	0.776	2.043	0.210	0.232	0.592	0.879	0.581	1.328	0.208	0.455	0.014	0.314	0.133	0.742
	rs10036748	0.226	0.222	0.89	1.027	0.595	1.773	0.231	0.185	0.293	1.324	0.816	2.149	0.218	0.235	0.669	0.908	0.600	1.373	0.215	0.455	0.015	0.328	0.139	0.774

† P values were calculated by Fisher's Exact test.

"+" represents patients with the subphenotype.

"-" represents patients without the subphenotype.

L95-U95 is the 95% confidence interval of each OR (odds ratio).

Table S4. Genotype distribution of the 18 SNPs in controls and different subphenotypes.

Gene	SNP	Genotype	Genotype counts			Genotype counts			Genotype counts			Genotype counts			Genotype counts			Genotype counts		
			Oneset age (<27, 84)	Control (504)	P†	Malar rash (+, 328)	Control (504)	P	Discoid rash (+, 73)	Control (504)	P	Photosensitivity (+, 78)	Control (504)	P	Oral ulcers (+, 77)	Control (504)	P	Arthritis (+, 341)	Control (504)	P
FCGR3A	rs7539036	TT/CT/CC	0/10/72	0/71/426	0.732	0/37/288	0/71/426	0.2466	0/13/60	0/71/426	0.4785	0/9/69	0/71/426	0.6003	0/9/68	0/71/426	0.7232	0/42/295	0/71/426	0.4721
	rs11581823	CC/CA/AA	0/14/68	1/91/385	0.7956	0/53/269	1/91/385	0.5225	0/10/62	1/91/385	0.4186	0/10/67	1/91/385	0.367	0/14/60	1/91/385	1	0/53/280	1/91/385	0.3195
FCGR2A	rs4656308	CC/CT/TT	2/24/57	12/121/365	0.6244	8/83/234	12/121/365	0.9082	3/14/56	12/121/365	0.4429	0/17/60	12/121/365	0.48	0/22/54	12/121/365	0.3647	9/88/240	12/121/365	0.803
	rs1801274	GG/GA/AA	10/42/31	68/202/227	0.2517	41/147/136	68/202/227	0.41	11/27/35	68/202/227	0.8302	10/37/30	68/202/227	0.4744	10/37/28	68/202/227	0.338	39/157/139	68/202/227	0.2028
TNFAIP3	rs5029924	TT/CT/CC	2/11/71	1/43/435	0.0327	1/53/272	1/43/435	0.003078	0/8/64	1/43/435	0.581	0/6/71	1/43/435	0.8552	1/6/68	1/43/435	0.3487	2/51/284	1/43/435	0.008865
	rs5029937	TT/GT/GG	2/11/70	1/43/458	0.02153	1/52/270	1/43/458	0.001692	0/8/65	1/43/458	0.5711	0/6/71	1/43/458	1	1/6/69	1/43/458	0.3394	2/50/284	1/43/458	0.005907
	rs2230926	GG/GT/TT	2/11/71	1/43/458	0.02239	1/53/273	1/43/458	0.001397	0/8/65	1/43/458	0.5711	0/6/72	1/43/458	1	1/6/70	1/43/458	0.3448	2/51/288	1/43/458	0.004878
	rs610604	GG/GT/TT	1/15/66	4/90/404	0.785	4/55/267	4/90/404	0.748	1/10/62	4/90/404	0.4042	1/16/61	4/90/404	0.5471	0/14/62	4/90/404	1	4/53/282	4/90/404	0.5642
TLR9	rs352162	GG/GA/AA	13/37/34	69/236/191	0.8035	54/140/134	69/236/191	0.338	11/34/28	69/236/191	0.9548	10/36/32	69/236/191	0.9289	10/31/36	69/236/191	0.3791	55/156/129	69/236/191	0.6576
	rs352140	AA/GA/GG	10/38/35	64/234/200	0.9444	49/139/138	64/234/200	0.4216	10/33/30	64/234/200	0.9535	9/36/32	64/234/200	0.9701	10/27/39	64/234/200	0.138	50/155/133	64/234/200	0.7318
	rs187084	CC/CT/TT	13/36/35	65/231/189	0.6784	53/137/136	65/231/189	0.2458	11/33/29	65/231/189	0.882	10/36/32	65/231/189	0.9563	10/30/36	65/231/189	0.3541	53/151/132	65/231/189	0.5832
TREX1	rs2242150	AA/CA/CC	12/32/39	62/208/227	0.7731	46/148/129	62/208/227	0.2595	12/30/31	62/208/227	0.5927	12/34/31	62/208/227	0.5706	15/27/33	62/208/227	0.202	46/154/135	62/208/227	0.3104
	rs3135941	CC/CT/TT	0/3/78	2/35/460	0.5109	0/22/295	2/35/460	0.7532	0/3/70	2/35/460	0.5869	0/1/75	2/35/460	0.1189	0/5/67	2/35/460	1	0/19/308	2/35/460	0.5143
ETS1	rs6590330	AA/GA/GG	21/37/26	36/236/227	1.37E-05	61/157/106	36/236/227	3.07E-07	10/34/28	36/236/227	0.1288	12/43/22	36/236/227	0.004607	7/37/33	36/236/227	0.7564	61/158/118	36/236/227	3.00E-06
	rs4937333	TT/CT/CC	23/39/21	54/255/188	0.0003019	76/164/83	54/255/188	7.82E-07	15/36/22	54/255/188	0.06291	20/43/15	54/255/188	0.0001625	13/41/23	54/255/188	0.1877	76/169/90	54/255/188	4.08E-06
TNIP1	rs13168551	TT/CT/CC	1/21/47	27/194/274	0.1043	11/90/161	27/194/274	0.2698	5/20/36	27/194/274	0.4644	1/27/34	27/194/274	0.455	4/20/35	27/194/274	0.6442	13/97/159	27/194/274	0.6411
	rs7708392	GG/GC/CC	1/20/47	29/195/265	0.04618	11/87/163	29/195/265	0.08698	6/17/38	29/195/265	0.115	1/23/34	29/195/265	0.4725	4/20/36	29/195/265	0.5784	13/97/159	29/195/265	0.4232
	rs10036748	CC/CT/TT	1/17/46	27/192/263	0.02653	12/88/157	27/192/263	0.237	5/19/36	27/192/263	0.3765	1/25/32	27/192/263	0.5181	4/18/34	27/192/263	0.4817	13/93/156	27/192/263	0.4423

Gene	SNP	Genotype	Genotype counts			Genotype counts			Genotype counts			Genotype counts			Genotype counts			Genotype counts		
			Serositis (+, 152)	Control (504)	P	Renal disorder (+, 273)	Control (504)	P	Neurologic disorder (+, 50)	Control (504)	P	Hematologic disorder (-, 370)	Control (504)	P	Immunologic disorder (+, 340)	Control (504)	P	Anti-nuclear antibody (+, 437)	Control (504)	P
FCGR3A	rs7539036	TT/CT/CC	0/13/138	0/71/426	0.07296	0/31/239	0/71/426	0.3166	0/3/46	0/71/426	0.1284	0/43/324	0/71/426	0.3093	0/44/292	0/71/426	0.6825	0/51/382	0/71/426	0.2846
	rs11581823	CC/CA/AA	0/28/119	1/91/385	1	0/40/228	1/91/385	0.2351	0/6/42	1/91/385	0.3922	0/55/307	1/91/385	0.1668	0/49/284	1/91/385	0.1407	0/68/360	1/91/385	0.2372
FCGR2A	rs4656308	CC/CT/TT	6/40/104	12/121/365	0.401	8/71/191	12/121/365	0.7201	2/11/36	12/121/365	0.6639	10/88/268	12/121/365	0.9566	7/83/246	12/121/365	0.9525	12/110/311	12/121/365	0.8439
	rs1801274	GG/GA/AA	8/68/73	68/202/227	0.01534	33/123/114	68/202/227	0.4239	8/22/19	68/202/227	0.6027	45/178/141	68/202/227	0.05461	43/160/131	68/202/227	0.1088	53/206/172	68/202/227	0.09152
TNFAIP3	rs5029924	TT/CT/CC	1/23/127	1/43/435	0.04473	1/35/236	1/43/435	0.1618	1/11/38	1/43/435	0.004128	2/54/310	1/43/435	0.01248	1/51/286	1/43/435	0.01158	2/62/370	1/43/435	0.0174
	rs5029937	TT/GT/GG	1/24/126	1/43/458	0.01304	1/34/234	1/43/458	0.1235	1/11/38	1/43/458	0.002091	2/54/309	1/43/458	0.00547	1/51/285	1/43/458	0.005157	2/61/369	1/43/458	0.01097
	rs2230926	GG/GT/TT	1/24/127	1/43/458	0.01682	1/35/236	1/43/458	0.1005	1/11/38	1/43/458	0.002091	2/55/312	1/43/458	0.004872	1/52/286	1/43/458	0.003815	2/62/372	1/43/458	0.009055
	rs610604	GG/GT/TT	1/26/124	4/90/404	0.961	4/42/225	4/90/404	0.465	1/6/43	4/90/404	0.2769	4/59/304	4/90/404	0.707	4/54/279	4/90/404	0.617	6/70/358	4/90/404	0.5174
TLR9	rs352162	GG/GA/AA	20/75/57	69/236/191	0.9419	48/125/100	69/236/191	0.4003	9/22/19	69/236/191	0.6801	51/163/155	69/236/191	0.5461	61/147/131	69/236/191	0.2312	69/192/175	69/236/191	0.5095
	rs352140	AA/GA/GG	18/73/60	64/234/200	0.9486	43/123/105	64/234/200	0.5192	9/20/21	64/234/200	0.4617	46/163/158	64/234/200	0.6892	55/145/137	64/234/200	0.3045	63/189/182	64/234/200	0.5406
	rs187084	CC/CT/TT	19/75/58	65/231/189	0.9407	46/123/102	65/231/189	0.412	9/22/19	65/231/189	0.6184	50/157/158	65/231/189	0.3772	60/143/134	65/231/189	0.1554	66/187/179	65/231/189	0.3933
TREX1	rs2242150	AA/CA/CC	21/66/62	62/208/227	0.6481	39/129/102	62/208/227	0.1072	6/22/21	62/208/227	0.9339	45/165/154	62/208/227	0.5752	47/145/143	62/208/227	0.6381	54/196/181	62/208/227	0.4931
	rs3135941	CC/CT/TT	0/9/137	2/35/460	0.912	0/14/249	2/35/460	0.4671	0/0/49	2/35/460	0.1166	0/21/336	2/35/460	0.4971	0/15/313	2/35/460	0.2003	0/24/399	2/35/460	0.378
ETS1	rs6590330	AA/GA/GG	26/73/51	36/236/227	0.0006257	52/125/93	36/236/227	1.95E-06	8/23/18	36/236/227	0.08571	65/186/116	36/236/227	2.19E-07	67/158/111	36/236/227	5.09E-08	78/211/144	36/236/227	1.72E-07
	rs4937333	TT/CT/CC	31/75/42	54/255/188	0.004248	61/138/69	54/255/188	7.79E-06	9/24/16	54/255/188	0.2866	78/195/91	54/255/188	2.01E-06	82/164/86	54/255/188	1.48E-07	92/225/111	54/255/188	1.60E-06
TNIP1	rs13168551	TT/CT/CC	6/52/60	27/194/274	0.6331	6/79/131	27/194/274	0.1961	2/15/27	27/194/274	0.8078	11/111/173	27/194/274	0.4554	9/101/165	27/194/274	0.2658	14/119/216	27/194/274	0.1595
	rs7708392	GG/GC/CC	6/48/62	29/195/265	0.9418	6/77/131	29/195/265	0.09308	2/15/27	29/195/265	0.7319	12/106/175	29/195/265	0.2489	9/97/168	29/195/265	0.08476	15/114/218	29/195/265	0.04247
	rs10036748	CC/CT/TT	7/46/61	27/192/263	0.9404	8/73/124	27/192/263	0.3118	3/13/26	27/192/263	0.4767	14/103/167	27/192/263	0.5303	11/94/161	27/192/263	0.2669	17/111/210	27/192/263	0.09449

Gene	SNP	Genotype	Genotype counts			Genotype counts			Genotype counts			Genotype counts			Genotype counts			Genotype counts		
			Oneset age (>= 27, 366)	Control (504)	P	Malar rash (-, 122)	Control (504)	P	Discoid rash (-, 377)	Control (504)	P	Photosensitivity (-, 372)	Control (504)	P	Oral ulcers (-, 373)	Control (504)	P	Arthritis (-, 109)	Control (504)	P
FCGR3A	rs7539036	TT/CT/CC	0/43/321	0/71/426	0.3099	0/16/105	0/71/426	0.8843	0/40/333	0/71/426	0.1246	0/44/324	0/71/426	0.3622	0/44/325	0/71/426	0.3622	0/11/98	0/71/426	0.2817
	rs11581823	CC/CA/AA	0/56/303	1/91/385	0.2481	0/17/102	1/91/385	0.4293	0/60/309	1/91/385	0.3614	0/60/304	1/91/385	0.4345	0/56/311	1/91/385	0.183	0/17/91	1/91/385	0.5865
FCGR2A	rs4656308	CC/CT/TT	10/89/264	12/121/365	0.946	4/30/87	12/121/365	0.7709	9/99/265	12/121/365	0.7649	12/96/261	12/121/365	0.5971	12/91/267	12/121/365	0.7358	3/25/81	12/121/365	0.9001
	rs1801274	GG/GA/AA	44/170/147	68/202/227	0.1728	13/65/42	68/202/227	0.03003	43/185/143	68/202/227	0.02635	44/175/148	68/202/227	0.12	44/175/150	68/202/227	0.144	15/55/39	68/202/227	0.1311
TNFAIP3	rs5029924	TT/CT/CC	0/53/309	1/43/435	0.0135	1/11/108	1/43/435	0.4969	2/56/316	1/43/435	0.009242	2/58/309	1/43/435	0.003713	1/58/312	1/43/435	0.004916	0/13/96	1/43/435	0.4838
	rs5029937	TT/GT/GG	0/52/310	1/43/458	0.009353	1/11/110	1/43/458	0.4896	2/55/315	1/43/458	0.005333	2/57/309	1/43/458	0.002021	1/57/311	1/43/458	0.002588	0/13/96	1/43/458	0.4036
	rs2230926	GG/GT/TT	0/53/312	1/43/458	0.008241	1/11/110	1/43/458	0.4896	2/56/318	1/43/458	0.004394	2/58/311	1/43/458	0.001505	1/58/313	1/43/458	0.002147	0/13/95	1/43/458	0.4002
	rs610604	GG/GT/TT	5/57/303	4/90/404	0.4784	2/17/102	4/90/404	0.2933	5/62/307	4/90/404	0.6208	5/56/308	4/90/404	0.3928	6/58/307	4/90/404	0.3705	2/19/87	4/90/404	0.5241
TLR9	rs352162	GG/GA/AA	57/162/146	69/236/191	0.6058	16/59/46	69/236/191	0.9698	59/165/152	69/236/191	0.5143	60/163/148	69/236/191	0.479	60/168/144	69/236/191	0.6104	15/43/51	69/236/191	0.2438
	rs352140	AA/GA/GG	54/158/152	64/234/200	0.5201	15/57/49	64/234/200	1	54/163/157	64/234/200	0.5783	55/160/155	64/234/200	0.488	54/169/148	64/234/200	0.7543	14/41/54	64/234/200	0.1672
	rs187084	CC/CT/TT	54/158/149	65/231/189	0.5251	14/57/48	65/231/189	0.9094	56/161/155	65/231/189	0.4366	57/158/152	65/231/189	0.3796	57/164/148	65/231/189	0.567	14/43/52	65/231/189	0.23
TREX1	rs2242150	AA/CA/CC	44/171/146	62/208/227	0.2513	10/55/56	62/208/227	0.4264	44/173/154	62/208/227	0.3648	44/169/154	62/208/227	0.473	41/176/152	62/208/227	0.2354	10/49/50	62/208/227	0.6271
	rs3135941	CC/CT/TT	0/22/333	2/35/460	0.5653	0/3/116	2/35/460	0.136	0/22/341	2/35/460	0.5339	0/24/336	2/35/460	0.6773	0/20/344	2/35/460	0.4111	0/6/103	2/35/460	0.7827
ETS1	rs6590330	AA/GA/GG	58/181/123	36/236/227	1.78E-05	18/61/43	36/236/227	0.01483	69/184/121	36/236/227	1.32E-07	67/175/127	36/236/227	1.02E-06	72/181/116	36/236/227	1.23E-08	18/60/31	36/236/227	0.0004214
	rs4937333	TT/CT/CC	71/193/94	54/255/188	4.32E-05	18/68/32	54/255/188	0.06002	79/196/93	54/255/188	2.37E-06	74/189/100	54/255/188	6.41E-05	81/191/92	54/255/188	1.06E-06	18/63/25	54/255/188	0.01033
TNIP1	rs13168551	TT/CT/CC	14/106/170	27/194/274	0.6871	4/37/56	27/194/274	0.8967	10/107/181	27/194/274	0.2093	14/100/183	27/194/274	0.2278	11/107/182	27/194/274	0.2572	2/30/58	27/194/274	0.2234
	rs7708392	GG/GC/CC	15/102/173	29/195/265	0.3437	5/35/57	29/195/265	0.7316	10/105/182	29/195/265	0.08057	15/99/186	29/195/265	0.1032	12/102/184	29/195/265	0.1006	3/25/61	29/195/265	0.0422
	rs10036748	CC/CT/TT	17/102/166	27/192/263	0.5412	6/31/55	27/192/263	0.5155	13/100/176	27/192/263	0.2266	17/94/180	27/192/263	0.1047	14/101/178	27/192/263	0.2432	5/26/56	27/192/263	0.1934

Gene	SNP	Genotype	Genotype counts			Genotype counts			Genotype counts			Genotype counts			Genotype counts			Genotype counts		
			Serositis (-, 298)	Control (504)	P	Renal disorder (-, 177)	Control (504)	P	Neurologic disorder (-, 400)	Control (504)	P	Hematologic disorder (-, 80)	Control (504)	P	Immunologic disorder (-, 110)	Control (504)	P	Anti-nuclear antibody (-, 13)	Control (504)	P
FCGR3A	rs7539036	TT/CT/CC	0/40/255	0/71/426	0.8326	0/22/154	0/71/426	0.6125	0/50/347	0/71/426	0.4919	0/10/69	0/71/426	0.8617	0/9/101	0/71/426	0.1178	0/2/11	0/71/426	1
	rs11581823	CC/CA/AA	0/42/252	1/91/385	0.1235	0/30/143	1/91/385	0.743	0/64/329	1/91/385	0.3264	0/15/64	1/91/385	1	0/21/87	1/91/385	1	0/2/11	1/91/385	1
FCGR2A	rs4656308	CC/CT/TT	6/73/217	12/121/365	0.9487	4/42/130	12/121/365	1	10/102/285	12/121/365	0.889	2/25/53	12/121/365	0.3796	5/30/75	12/121/365	0.2926	0/3/10	12/121/365	1
	rs1801274	GG/GA/AA	46/144/105	68/202/227	0.01999	21/89/64	68/202/227	0.0557	46/190/159	68/202/227	0.08212	9/34/37	68/202/227	0.8657	11/52/47	68/202/227	0.3722	1/6/6	68/202/227	0.9305
TNFAIP3	rs5029924	TT/CT/CC	1/41/253	1/43/435	0.05399	1/29/144	1/43/435	0.008483	1/53/342	1/43/435	0.06518	0/10/70	1/43/435	0.4062	1/13/94	1/43/435	0.211	0/2/10	1/43/435	0.3204
	rs5029937	TT/GT/GG	1/39/254	1/43/458	0.06452	1/29/146	1/43/458	0.005266	1/52/342	1/43/458	0.04146	0/9/71	1/43/458	0.486	1/12/95	1/43/458	0.2003	0/2/11	1/43/458	0.3345
	rs2230926	GG/GT/TT	1/40/256	1/43/458	0.04696	1/29/147	1/43/458	0.006103	1/53/345	1/43/458	0.04256	0/9/71	1/43/458	0.486	1/12/97	1/43/458	0.2322	0/2/11	1/43/458	0.3345
	rs610604	GG/GT/TT	5/46/245	4/90/404	0.3593	2/30/144	4/90/404	0.7951	5/66/326	4/90/404	0.664	2/13/65	4/90/404	0.3021	2/18/90	4/90/404	0.4806	0/2/11	4/90/404	1
TLR9	rs352162	GG/GA/AA	50/124/123	69/236/191	0.24	22/74/80	69/236/191	0.2765	61/177/161	69/236/191	0.6069	19/36/25	69/236/191	0.07487	9/52/49	69/236/191	0.216	1/7/5	69/236/191	0.9298
	rs352140	AA/GA/GG	46/123/127	64/234/200	0.2803	21/73/82	64/234/200	0.3294	55/176/166	64/234/200	0.7205	18/33/29	64/234/200	0.08567	9/51/50	64/234/200	0.3326	1/7/5	64/234/200	1
	rs187084	CC/CT/TT	48/119/126	65/231/189	0.1441	21/71/82	65/231/189	0.1752	58/172/165	65/231/189	0.4811	17/37/26	65/231/189	0.1718	7/51/50	65/231/189	0.09149	1/7/5	65/231/189	1
TREX1	rs2242150	AA/CA/CC	35/137/123	62/208/227	0.4526	17/74/83	62/208/227	0.6606	50/181/164	62/208/227	0.4385	11/38/31	62/208/227	0.4918	9/58/42	62/208/227	0.08959	2/7/4	62/208/227	0.5727
	rs3135941	CC/CT/TT	0/16/274	2/35/460	0.4541	0/11/162	2/35/460	0.9243	0/25/362	2/35/460	0.611	0/4/75	2/35/460	0.7287	0/10/98	2/35/460	0.6098	0/1/12	2/35/460	1
ETS1	rs6590330	AA/GA/GG	53/145/98	36/236/227	2.90E-06	27/93/56	36/236/227	0.0004574	71/195/131	36/236/227	3.24E-07	14/32/33	36/236/227	0.0157	12/60/38	36/236/227	0.07626	1/7/5	36/236/227	0.8312
	rs4937333	TT/CT/CC	63/157/73	54/255/188	8.53E-06	33/94/46	54/255/188	0.003412	85/208/99	54/255/188	1.15E-06	16/37/24	54/255/188	0.0536	12/68/29	54/255/188	0.06942	2/7/4	54/255/188	0.7154
TNIP1	rs13168551	TT/CT/CC	9/75/157	27/194/274	0.04113	9/48/86	27/194/274	0.4483	13/112/190	27/194/274	0.3576	4/16/44	27/194/274	0.07457	6/26/52	27/194/274	0.325	1/8/1	27/194/274	0.009708
	rs7708392	GG/GC/CC	10/74/158	29/195/265	0.01725	10/45/89	29/195/265	0.1665	14/107/193	29/195/265	0.123	4/16/45	29/195/265	0.05042	7/25/52	29/195/265	0.1626	1/8/2	29/195/265	0.04707
	rs10036748	CC/CT/TT	11/73/151	27/192/263	0.0464	10/46/88	27/192/263	0.2211	15/106/186	27/192/263	0.2563	4/16/45	27/192/263	0.04956	7/25/51	27/192/263	0.1789	1/8/2	27/192/263	0.04282

Gene	SNP	Genotype	Genotype counts			Genotype counts			Genotype counts			Genotype counts			Genotype counts			Genotype counts		
			Oneset age (<27, 84)	Oneset age (>= 27, 366)	P	Malar rash (+, 328)	Malar rash (-, 122)	P	Discoid rash (+, 73)	Discoid rash (-, 377)	P	Photosensitivity (+, 78)	Photosensitivity (-, 372)	P	Oral ulcers (+, 77)	Oral ulcers (-, 373)	P	Arthritis (+, 341)	Arthritis (-, 109)	P
FCGR3A	rs7539036	TT/CT/CC	0/10/72	0/43/321	1	0/37/288	0/16/105	0.6224	0/13/60	0/40/333	0.1113	0/9/69	0/44/324	1	0/9/68	0/44/325	1	0/42/295	0/11/98	0.6104
	rs11581823	CC/CA/AA	0/14/68	0/56/303	0.7389	0/53/269	0/17/102	0.6606	0/10/62	0/60/309	0.7256	0/10/67	0/60/304	0.4973	0/14/60	0/56/311	0.485	0/53/280	0/17/91	1
FCGR2A	rs4656308	CC/CT/TT	2/24/57	10/89/264	0.6756	8/83/234	4/30/87	0.8816	3/14/56	9/99/265	0.2954	0/17/60	12/96/261	0.2154	0/22/54	12/91/267	0.2573	9/88/240	3/25/81	0.8051
	rs1801274	GG/GA/AA	10/42/31	44/170/147	0.8445	41/147/136	13/65/42	0.2613	11/27/35	43/185/143	0.1139	10/37/30	44/175/148	0.9372	10/37/28	44/175/150	0.819	39/157/139	15/55/39	0.5396
TNFAIP3	rs5029924	TT/CT/CC	2/11/71	0/53/309	0.05174	1/53/272	1/11/108	0.09038	0/8/64	2/56/316	0.6244	0/6/71	2/58/309	0.1406	1/6/68	1/58/312	0.07844	2/51/284	0/13/96	0.6785
	rs5029937	TT/GT/GG	2/11/70	0/52/310	0.0596	1/52/270	1/11/110	0.09183	0/8/65	2/55/315	0.6266	0/6/71	2/57/309	0.1605	1/6/69	1/57/311	0.07942	2/50/284	0/13/96	0.7311
	rs2230926	GG/GT/TT	2/11/71	0/53/312	0.04655	1/53/273	1/11/110	0.09169	0/8/65	2/56/318	0.6257	0/6/72	2/58/311	0.1413	1/6/70	1/58/313	0.08039	2/51/288	0/13/95	0.7284
	rs610604	GG/GT/TT	1/15/66	5/57/303	0.8462	4/55/267	2/17/102	0.6562	1/10/62	5/62/307	0.8403	1/16/61	5/56/308	0.4193	0/14/62	6/58/307	0.6267	4/53/282	2/19/87	0.6673
TLR9	rs352162	GG/GA/AA	13/37/34	57/162/146	1	54/140/134	16/59/46	0.4918	11/34/28	59/165/152	0.9401	10/36/32	60/163/148	0.791	10/31/36	60/168/144	0.4404	55/156/129	15/43/51	0.2682
	rs352140	AA/GA/GG	10/38/35	54/158/152	0.8295	49/139/138	15/57/49	0.6569	10/33/30	54/163/157	0.9842	9/36/32	55/160/155	0.7834	10/27/39	54/169/148	0.1786	50/155/133	14/41/54	0.1719
	rs187084	CC/CT/TT	13/36/35	54/158/149	0.9861	53/137/136	14/57/48	0.4001	11/33/29	56/161/155	0.9542	10/36/32	57/158/152	0.812	10/30/36	57/164/148	0.5352	53/151/132	14/43/52	0.3031
TREX1	rs2242150	AA/CA/CC	12/32/39	44/171/146	0.3304	46/148/129	10/55/56	0.1942	12/30/31	44/173/154	0.449	12/34/31	44/169/154	0.6809	15/27/33	41/176/152	0.05968	46/154/135	10/49/50	0.3838
	rs3135941	CC/CT/TT	0/3/78	0/22/333	0.5954	0/22/295	0/3/116	0.1039	0/3/70	0/22/341	0.782	0/1/75	0/24/336	0.09854	0/5/67	0/20/344	0.5837	0/19/308	0/6/103	1
ETS1	rs6590330	AA/GA/GG	21/37/26	58/181/123	0.1641	61/157/106	18/61/43	0.6108	10/34/28	69/184/121	0.4778	12/43/22	67/175/127	0.4223	7/37/33	72/181/116	0.0369	61/158/118	18/60/31	0.3262
	rs4937333	TT/CT/CC	23/39/21	71/193/94	0.2744	76/164/83	18/68/32	0.1627	15/36/22	79/196/93	0.675	20/43/15	74/189/100	0.2573	13/41/23	81/191/92	0.5202	76/169/90	18/63/25	0.2597
TNIP1	rs13168551	TT/CT/CC	1/21/47	14/106/170	0.2718	11/90/161	4/37/56	0.7742	5/20/36	10/107/181	0.2471	1/27/34	14/100/183	0.2841	4/20/35	11/107/182	0.5091	13/97/159	2/30/58	0.5133
	rs7708392	GG/GC/CC	1/20/47	15/102/173	0.2664	11/87/163	5/35/57	0.7605	6/17/38	10/105/182	0.06459	1/23/34	15/99/186	0.4309	4/20/36	12/102/184	0.6563	13/97/159	3/25/61	0.2919
	rs10036748	CC/CT/TT	1/17/46	17/102/166	0.1064	12/88/157	6/31/55	0.7648	5/19/36	13/100/176	0.4389	1/25/32	17/94/180	0.2057	4/18/34	14/101/178	0.7002	13/93/156	5/26/56	0.6135

Gene	SNP	Genotype	Genotype counts			Genotype counts			Genotype counts			Genotype counts			Genotype counts			Genotype counts		
			Serositis (+, 152)	Serositis (-, 298)	P	Renal disorder (+, 273)	Renal disorder (-, 177)	P	Neurologic disorder (+, 50)	Neurologic disorder (-, 400)	P	Hematologic disorder (-, 370)	Hematologic disorder (-, 80)	P	Immunologic disorder (+, 340)	Immunologic disorder (-, 110)	P	Anti-nuclear antibody (+, 437)	Anti-nuclear antibody (-, 13)	P
FCGR3A	rs7539036	TT/CT/CC	0/13/138	0/40/255	0.1635	0/31/239	0/22/154	0.766	0/3/46	0/50/347	0.2442	0/43/324	0/10/69	0.8481	0/44/292	0/9/101	0.2337	0/51/382	0/2/11	0.6591
	rs11581823	CC/CA/AA	0/28/119	0/42/252	0.2144	0/40/228	0/30/143	0.5072	0/6/42	0/64/329	0.6754	0/55/307	0/15/64	0.3985	0/49/284	0/21/87	0.2883	0/68/360	0/2/11	1
FCGR2A	rs4656308	CC/CT/TT	6/40/104	6/73/217	0.3804	8/71/191	4/42/130	0.7934	2/11/36	10/102/285	0.6376	10/88/268	2/25/53	0.4319	7/83/246	5/30/75	0.2766	12/110/311	0/3/10	1
	rs1801274	GG/GA/AA	8/68/73	46/144/105	0.0009345	33/123/114	21/89/64	0.4891	8/22/19	46/190/159	0.608	45/178/141	9/34/37	0.4753	43/160/131	11/52/47	0.6955	53/206/172	1/6/6	0.9251
TNFAIP3	rs5029924	TT/CT/CC	1/23/127	1/41/253	0.8012	1/35/236	1/29/144	0.4042	1/11/38	1/53/342	0.05252	2/54/310	0/10/70	0.8153	1/51/286	1/13/94	0.3713	2/62/370	0/2/10	0.7033
	rs5029937	TT/GT/GG	1/24/126	1/39/254	0.5348	1/34/234	1/29/146	0.4048	1/11/38	1/52/342	0.04305	2/54/309	0/9/71	0.6513	1/51/285	1/12/95	0.2738	2/61/369	0/2/11	1
	rs2230926	GG/GT/TT	1/24/127	1/40/256	0.6178	1/35/236	1/29/147	0.4643	1/11/38	1/53/345	0.0515	2/55/312	0/9/71	0.6503	1/52/286	1/12/97	0.2782	2/62/372	0/2/11	1
	rs610604	GG/GT/TT	1/26/124	5/46/245	0.7249	4/42/225	2/30/144	0.9033	1/6/43	5/66/326	0.4893	4/59/304	2/13/65	0.5119	4/54/279	2/18/90	0.8029	6/70/358	0/2/11	1
TLR9	rs352162	GG/GA/AA	20/75/57	50/124/123	0.2862	48/125/100	22/74/80	0.1275	9/22/19	61/177/161	0.8619	51/163/155	19/36/25	0.05075	61/147/131	9/52/49	0.03812	69/192/175	1/7/5	0.8056
	rs352140	AA/GA/GG	18/73/60	46/123/127	0.3507	43/123/105	21/73/82	0.2142	9/20/21	55/176/166	0.6767	46/163/158	18/33/29	0.07679	55/145/137	9/51/50	0.09827	63/189/182	1/7/5	0.798
	rs187084	CC/CT/TT	19/75/58	48/119/126	0.1983	46/123/102	21/71/82	0.1075	9/22/19	58/172/165	0.7561	50/157/158	17/37/26	0.1018	60/143/134	7/51/50	0.01037	66/187/179	1/7/5	0.8011
TREX1	rs2242150	AA/CA/CC	21/66/62	35/137/123	0.7728	39/129/102	17/74/83	0.08757	6/22/21	50/181/164	0.9771	45/165/154	11/38/31	0.8299	47/145/143	9/58/42	0.1215	54/196/181	2/7/4	0.67
	rs3135941	CC/CT/TT	0/9/137	0/16/274	0.8283	0/14/249	0/11/162	0.6773	0/0/49	0/25/362	0.09552	0/21/336	0/4/75	1	0/15/313	0/10/98	0.09215	0/24/399	0/1/12	0.541
ETS1	rs6590330	AA/GA/GG	26/73/51	53/145/98	0.9815	52/125/93	27/93/56	0.3592	8/23/18	71/195/131	0.8992	65/186/116	14/32/33	0.1855	67/158/111	12/60/38	0.08064	78/211/144	1/7/5	0.807
	rs4937333	TT/CT/CC	31/75/42	63/157/73	0.7467	61/138/69	33/94/46	0.6668	9/24/16	85/208/99	0.54	78/195/91	16/37/24	0.5277	82/164/86	12/68/29	0.00508	92/225/111	2/7/4	0.8703
TNIP1	rs13168551	TT/CT/CC	6/52/60	9/75/157	0.03272	6/79/131	9/48/86	0.264	2/15/27	13/112/190	1	11/111/173	4/16/44	0.1069	9/101/165	6/26/52	0.2143	14/119/216	1/8/1	0.002212
	rs7708392	GG/GC/CC	6/48/62	10/74/158	0.09029	6/77/131	10/45/89	0.1408	2/15/27	14/107/193	1	12/106/175	4/16/45	0.1787	9/97/168	7/25/52	0.1208	15/114/218	1/8/2	0.008045
	rs10036748	CC/CT/TT	7/46/61	11/73/151	0.1514	8/73/124	10/46/88	0.3777	3/13/26	15/106/186	0.6649	14/103/167	4/16/45	0.1766	11/94/161	7/25/51	0.2439	17/111/210	1/8/2	0.008819

† P values were calculated by Fisher's Exact test.
"+" represents patients with the subphenotype.
"-" represents patients without the subphenotype.
